# Synthesis and X-ray characterization of 15- and 16-vertex *closo*-carboranes

**DOI:** 10.1038/s41467-020-19661-5

**Published:** 2020-11-23

**Authors:** Fangrui Zheng, Tsz Hin Yui, Jiji Zhang, Zuowei Xie

**Affiliations:** Department of Chemistry and State Key Laboratory of Synthetic Chemistry, The Chinese University of Hong Kong, Shatin, New Territories, Hong Kong, China

**Keywords:** Synthetic chemistry methodology, Organometallic chemistry, Supramolecular chemistry

## Abstract

Carboranes are a class of carbon-boron molecular clusters with three-dimensional aromaticity, and inherent robustness. These endowments enable carboranes as valuable building blocks for applications ranging from functional materials to pharmaceuticals. Thus, the chemistry of carboranes has received tremendous research interest, and significant progress has been made in the past decades. However, many attempts to the synthesis of carboranes with more than 14 vertices had been unsuccessful since the report of a 14-vertex carborane in 2005. The question arises as to whether these long sought-after molecules exist. We describe in this article the synthesis and structural characterization of 15- and 16-vertex *closo*-carboranes as well as 16-vertex ruthenacarborane. Such a success relies on the introduction of silyl groups to both cage carbons, stabilizing the corresponding *nido*-carborane dianions and promoting the capitation reaction with HBBr_2_·SMe_2._ This work would shed some light on the preparation of carboranes with 17 vertices or more, and open the door for studying supercarborane chemistry.

## Abstract (web summary)

The synthesis of *closo*-carboranes with more than 14 vertexes is challenging, and no examples have been reported to date. Herein, the authors present the long-sought 15- and 16-vertex closo-carboranes, in which the introduction of silyl groups to the two cage carbons is crucial; this finding might enable the synthesis of even larger carborane analogs in the future.

## Introduction

Carboranes are a class of carbon-boron molecular clusters with 3D aromaticity. They are finding a wide variety of applications as functional building blocks in the supramolecular design, medicine, catalysts, nanomaterials, and more^1,2^. These applications would be benefitted from higher nuclearity clusters containing large numbers of boron atoms. Thus, the chemistry of carboranes has attracted tremendous attention, particularly in the catalytic selective functionalization of carboranes^1,3–11^ and polyhedral expansion for the synthesis of supercarboranes (carboranes with more than 12 vertices)^12–14^.

In contrast to the extensively studied icosahedral *o*-carboranes that have dominated the carborane chemistry for over half a century^1,15,16^, has considerable progress been made in the chemistry of supercarboranes only since 2003^12^. Such a breakthrough relied on the use of relatively weaker reducing compounds, CAd (carbon atoms-adjacent) *nido*-carborane dianions or *arachno*-carborane tetraanions^17^, as starting materials for polyhedral expansion reactions^18,19^. A number of 13- and 14-vertex *closo*-carboranes were prepared and structurally characterized^20–29^. They showed some unique characteristics. For example, a 13-vertex *closo*-carborane accepted one electron to give a stable carborane radical anion with [2*n* + 3] framework electrons^30,31^; it also reacted with various nucleophiles to afford the cage carbon and/or cage boron extrusion products *closo*-CB_11_^−^, *nido*-CB_10_^−^, *closo*-CB_10_^−^, and *closo*-C_2_B_10_, depending on the nature of the nucleophiles^32–35^. Insertion of a metal fragment into a 14-vertex CAd *nido*-carborane was successful, resulting in the isolation and structural characterization of a 15-vertex metallacarborane 1,4-(CH_2_)_3_-7-(*p*-cymene)-7,1,4-RuC_2_B_12_H_12_^23^. However, the reactions of CAd [*nido*-(CH_2_)_3_C_2_B_12_H_12_]Na_2_ with haloboranes RBX_2_ under different reaction conditions did not afford any 15-vertex *closo*-carborane, rather generated a structural isomer of CAd 14-vertex *closo*-carborane along with some inseparable very polar boron-containing species^23^. These results indicated that redox reaction proceeded in the system, in which *nido*-carborane was oxidized to the corresponding *closo*-species. To suppress such redox reactions and promote the capping reaction, a 14-vertex *nido*-carborane with lower reducing power is necessary.

It was documented that silyl groups can stabilize carboanions and the stabilization is approximately additive for the number of silyl groups^36^. We then speculated that the introduction of silyl groups to both cage carbon vertices should significantly stabilize the corresponding *nido*-carborane dianions, so as to lower their reducing power. On the other hand, our experimental and theoretical results indicate that CAp (carbon atoms-apart) *closo*-carboranes are thermodynamically more stable than the corresponding CAd *closo-*species^37^. For instance, CAp 15-vertex carborane is calculated to be thermodynamically more stable over CAd isomer by 22.4 kcal/mol^37^. In view of the role of silyl groups and extra stability of CAp supercarboranes, we initiated a research program to prepare supercarboranes using 1,2-(R_3_Si)_2_-1,2-C_2_B_10_H_10_ as starting materials.

In this work, we report the synthesis and structural characterization of CAp 15- and 16-vertex *closo*-carboranes as well as 16-vertex ruthenacarborane. The results indicate that they are thermodynamically very stable.

## Results and discussion

### 15-Vertex *closo*-carborane

**Fig. 1:**
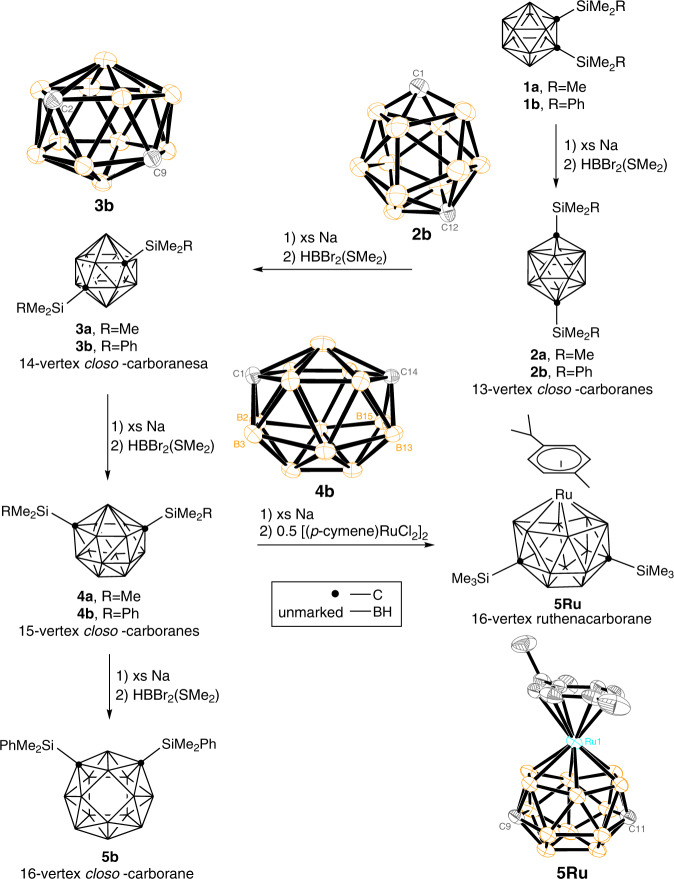
Synthesis of 13- to 16-vertex *closo*-carboranes and 16-vertex ruthenacarborane. Synthetic route along with the molecular structures of 13-vertex *closo*-carborane (**2b**), 14-vertex *closo*-carborane (**3b**), 15-vertex *closo*-carborane (**4b**), and 16-vertex ruthenacarborane (**5Ru**). For clarity, in the X-ray structures, all silyl moieties and hydrogen atoms have been omitted. Orange, B; grey, C.

Reduction of 1,2-(RMe_2_Si)_2_-1,2-C_2_B_10_H_10_ [R = Me (**1a**), Ph (**1b**)] with an excess amount of finely cut sodium metal in THF at room temperature gave presumably CAp *nido*-carborane salts [{7,9-(RMe_2_Si)_2_-7,9-C_2_B_10_H_10_}{Na_2_(THF)_*x*_}]^38,39^. Treatment of these salts with 2 equiv. of HBBr_2_·SMe_2_ in dimethoxyethane (DME) from −78 to 25 °C afforded, after column chromatographic separation, CAp 13-vertex *closo*-carboranes 1,12-(RMe_2_Si)_2_-1,12-C_2_B_11_H_11_ [R = Me (**2a**), Ph (**2b**)] in 20–29% isolated yields. Their ^11^B NMR spectra exhibited a pattern of 1:2:1:3:3:1 ranging from *δ* = 15 to −17 ppm. Single-crystal X-ray analyses confirm that the cage carbons are located in 1- and 12-positions, respectively, in both **2a** and **2b** (Fig. 1).

Treatment of **2** with an excess amount of finely cut sodium metal in THF at room temperature, followed by the reaction with 3 equiv. of HBBr_2_·SMe_2_ in CH_2_Cl_2_ (DCM) from −78 to 25 °C afforded, after column chromatographic separation, CAp 14-vertex *closo*-carboranes 2,9-(RMe_2_Si)_2_-2,9-C_2_B_12_H_12_ [R = Me (**3a**), Ph (**3b**)] in 15–20% isolated yields (Fig. 1). The ^11^B NMR spectra of **3** exhibited a pattern of 2:4:4:2, ranging from *δ* = 10 to −20 ppm. Their solid-state structures were further confirmed by single-crystal X-ray analyses, showing that they adopt a bicapped hexagonal antiprismatic geometry as that of 2,9-Me_2_-2,9-C_2_B_12_H_12_^27^, with two cage carbons being located at 2- and 9-positions.

Compounds **3** were treated with an excess amount of finely cut sodium metal in THF, followed by the reaction with 3 equiv. of HBBr_2_·SMe_2_ from −78 to 25 °C in CH_2_Cl_2_ gave, after column chromatographic separation, 1,14-(RMe_2_Si)_2_-1,14-C_2_B_13_H_13_ [R = Me (**4a**), Ph (**4b**)] in 3–8% isolated yields (Fig. 1). Their ^11^B NMR spectra showed a pattern of 2:4:4:1:2 spanning the range *δ* = 16 to −21 ppm. Single-crystal X-ray analyses show that **4b** adopts a *closo* structure with 26 triangulated faces and 32 framework electrons. It has an approximate *D*_3h_ symmetry with a *C*_3_ axis passing through the centers of C(1)B(2)B(3) and B(13)C(14)B(15) trigonal planes if omitting two dimethylphenylsilyl (DMPS) groups and the differentia among boron and carbon atoms. The two 6-coordinate cage carbons are located at 1-, 14-positions, respectively, which is the second stable CAp isomer predicted by DFT results^37^. There are three 7-coordinate [B(7), B(8) and B(9)], and ten 6-coordinate boron atoms.

### 16-Vertex ruthenacarborane

Reduction of **4a** with an excess amount of finely cut sodium metal in THF at room temperature, followed by interaction with 0.5 equiv. of [(*p*-cymene)RuCl_2_]_2_ in THF from −30 to 25 °C afforded, after column chromatographic separation, a 16-vertex ruthenacarborane 9,11-(Me_3_Si)_2_-1-(*p*-cymene)-1,9,11-RuC_2_B_13_H_13_ (**5Ru**) in 38% isolated yield as yellow crystals. Its ^11^B NMR spectrum exhibited a 2:2:2:1:4:2 pattern in the range *δ* = 0.1 to −28.2 ppm, which was very different from that of **4a**. Single-crystal X-ray analyses revealed that **5Ru** adopts a *closo* structure with 26 triangular and one rhombus faces, in which the Ru atom is *η*^6^-bonded to a six-membered B_6_ ring with an average Ru-B distance of 2.274(3) Å (Fig. 1)^40,41^. This measured value is very close to that of 2.247(3) Å in 1,4-(CH_2_)_3_-7-(*p*-cymene)-7,1,4-RuC_2_B_12_H_12_^23^. The two 6-coordinate cage carbons are located in 9- and 11-positions, respectively. Careful examination of the X-ray structures of **4b** and **5Ru** clearly indicated that significant cage rearrangement occurred during the reaction. A DSD (diamond-square-diamond) mechanism may be involved in such a skeletal rearrangement^42^.

### 16-Vertex *closo*-carborane

Treatment of **4b** with an excess amount of finely cut sodium in THF at room temperature for 3 days, followed by reaction with 3 equiv. of HBBr_2_·SMe_2_ in CH_2_Cl_2_ afforded, after column chromatographic separation, a CAp 16-vertex *closo*-carborane **5b** in 26% isolated yield as colorless crystals (Fig. 1). It is stable in air and soluble in common organic solvents such as *n*-hexane, acetone, CH_2_Cl_2_, and CHCl_3_. Attempts to prepare *closo*-C_2_B_14_H_16_ by removing two silyl groups from **5b** were not successful.

**Fig. 2:**
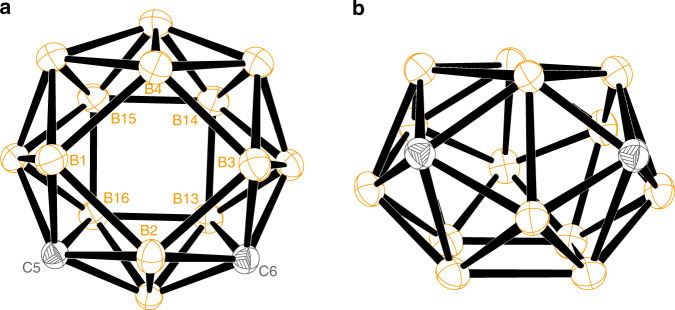
Molecular structure of 16-vertex *closo*-carborane 5b in different view angles. **a** View down from the B(1)B(2)B(3)B(4) face. **b** View down from the C(5) and C(6) atoms. Both silyl moieties and hydrogen atoms are omitted for clarity. Orange, B; grey, C.

The ^11^B NMR spectrum of **5b** displayed a pattern of 1:4:4:3:2 spanning the range *δ* = 14.3 to −18.3 ppm. Its solid-state structure was confirmed by single-crystal X-ray analyses (Fig. 2). Compound **5b** adopts a *closo* structure with 34 framework electrons, 24 triangular and two rhombus faces, in which the two 6-coordinate cage carbons are located in the 5, 6-positions, respectively. If omitting two DMPS groups and the differentia among boron and carbon atoms, **5b** has an approximate *D*_4d_ symmetry with a *C*_4_ axis passing through the centers of B(1)B(2)B(3)B(4) and B(13)B(14)B(15)B(16) faces (Fig. 2a). Such a geometry is predicted to be the third most stable isomer by DFT calculation^37^. It could also be viewed as a square-crown-square type of structure with two cage carbons located on the crown (Fig. 2b).

**Fig. 3:**
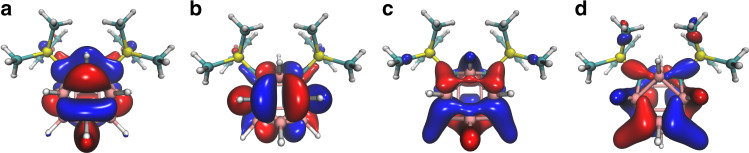
Selected molecular orbitals of 16-vertex *closo*-carborane 5b calculated at the B3LYP/6-31G(d,p) level of theory. **a** LUMO + 1. **b** LUMO. **c** HOMO-4. **d** HOMO-5.

It has been well documented that *closo*-carboranes/*closo*-polyhedral boranes are three-dimensional aromatic molecules, which have electrons delocalized through a set of orbitals tangential to the polyhedral surface^43,44^. The structural features of **4b** and **5b** were confirmed by density functional theory (DFT) calculations at the B3LYP level of theory (see computational details in the Supplementary Information and cartesian coordinates in Supplementary Data 1 and 2). The HOMO (highest occupied molecular orbital) is composed of the contribution of the π orbitals of the phenyl rings from the SiMe_2_Ph groups (see Supplementary Figs. 8 and 9). The LUMO (lowest unoccupied molecular orbitals) exhibits anti-bonding characters of cage tangential orbitals. Figure 3 shows the LUMO, LUMO + 1, HOMO-4, and HOMO-5 orbitals of **5b**.

In summary, we have prepared and fully characterized 15- and 16-vertex *closo*-carboranes as well as 16-vertex ruthenacarborane. The results indicate that they are thermodynamically very stable. Such a success relies on the introduction of silyls onto the two cage carbons, stabilizing the corresponding *nido*-carborane dianions, and thus facilitating the capitation reaction. Following this strategy, if silyl groups would be introduced to cage boron atoms, it is anticipated that carboranes with 17 vertices or more would be prepared in the future as they are calculated to be thermodynamically more stable than 13- and 15-vertex *closo-*carboranes^37^. On the other hand, it is further anticipated that these superclusters may find applications in coordination chemistry/materials science in view of the recent development in this field^45–48^.

## Methods

The synthetic protocol and the characterization of compounds **1a**–**4a**, **1b**–**5b**, and **5Ru** can be found in the Supplementary Information.

## Supplementary information

Supplementary Information

Description of Additional Supplementary Files

Supplementary Data 1

Supplementary Data 2

## Data Availability

CCDC 1997899 (**2a**), 1997900 (**2b**), 1997901 (**3a**), 1997902 (**3b**), 1997903 (**4b**), 1997904 (**5b**), and 1997905 (**5Ru**) contain the supplementary crystallographic data for this paper. These data can be obtained free of charge from The Cambridge Crystallographic Data Centre via www.ccdc.cam.ac.uk/data_request/cif.
