# Supplementary material for: Synthesis and X-ray characterization of 15- and 16-vertex closo-carboranes
Source: Nat Commun. 2020 Nov 23;11:5943. doi: 10.1038/s41467-020-19661-5 (PMC7683565; doi:10.1038/s41467-020-19661-5)
Supplement: Supplementary file 1 — Supplementary Information [file 41467_2020_19661_MOESM1_ESM.pdf]

Supplementary Information

Synthesis and X-ray characterization of 15- and 16-vertex *closo*-carboranes

Fangrui Zheng[1,†], Tsz Hin Yui[1,†], Jiji Zhang[1] & Zuowei Xie[1,★]

[1] *Department of Chemistry and State Key Laboratory of Synthetic Chemistry, The Chinese University of Hong Kong, Shatin, New Territories, Hong Kong, China*

★e-mail: zxie@cuhk.edu.hk

†These authors contributed equally

Table of Contents

Supplementary Method S 2

General Procedure S 2

Experimental Section S 2

Computational Detail S 1 0

NMR Spectra of New Compounds S13

Supplementary Reference S25

Supplementary Method

General Procedure

Unless otherwise noted, all experiments were performed under an atmosphere of dry argon with the rigid exclusion of air and moisture using standard Schlenk or cannula techniques, or in a glovebox. All organic solvents were refluxed over sodium benzophenone ketyl for several days and freshly distilled prior to use. CH_2Cl_2 was refluxed over CaH_2 for several days and distilled immediately before use. All other chemicals were purchased from either Aldrich or Acros Chemical Company and used as received unless otherwise noted. The ^1H and ^{13}C NMR spectra were recorded on a Bruker DPX 400 spectrometer at 400 and 100 MHz, respectively. The ^{11}B NMR spectra were recorded on a Bruker DPX 300 spectrometer at 96 MHz or a Bruker DPX 400 spectrometer at 128 MHz, respectively. All chemical shifts were reported in δ units with references to the residual protons or carbons of the deuterated solvents for proton or carbon chemical shifts, and to external $BF_3 \cdot OEt_2$ (0.0 ppm) for boron chemical shifts. Mass spectra were recorded on a Thermo Finnigan MAT 95 XL spectrometry.

Experimental Section

Preparation of 1,2-TMS$_2$-1,2-C$_2$B$_{10}$H$_{10}$ (1a). To a THF (20 mL) solution of *o*-carborane (4.61 g, 32.0 mmol) was slowly added ^{n}BuLi (40.0 mL of 1.6 M in *n*-hexane, 64.0 mmol) at 0 °C, and the reaction mixture was stirred at this temperature for 1 h, and then at room temperature for another 1 h. To the resultant solution was slowly added chlorotrimethylsilane (8.5 mL, 66.7 mmol) at 0 °C, the mixture was stirred at this temperature for 1 h, and then at room temperature overnight. After hydrolysis with water (20 mL) and extraction with diethyl ether (3 x 20 mL), the organic solutions were combined, dried with $MgSO_4$ and concentrated to dryness in vacuo. The residue was recrystallized from acetone to afford **1a** (7.57 g, 82%) as colorless crystals. ^1H NMR ($CDCl_3$): δ 0.32 (s, 18H, Si(C*H*$_3$)$_3$). $^{13}C\{^1H\}$ NMR ($CDCl_3$): δ 1.6 (Si(*C*H$_3$)$_3$), 74.7 (cage *C*). $^{11}B\{^1H\}$ NMR ($CDCl_3$): δ 1.7 (2B), -5.9 (2B), -9.0 (4B), -11.2 (2B). HRMS: *m/z* calcd for $C_8H_{28}B_{10}Si_2$ $[M]^+$: 288.2743. Found: 288.2743. This is a known compound.[1]

Preparation of 1,2-DMPS$_2$-1,2-C$_2$B$_{10}$H$_{10}$ (1b). To a THF (20 mL) solution of *o*-carborane (4.61 g, 32.0 mmol) was slowly added ^{n}BuLi (40.0 mL of 1.6 M in *n*-hexane, 64.0 mmol) at 0°C, the mixture was stirred at this temperature for 1 h, and then at room temperature overnight. Chlorodimethylphenylsilane (11.2 mL, 66.7 mmol) was then slowly added to the above suspension at 0°C, the mixture was stirred at this temperature for 1 h, and then at room temperature overnight. After hydrolysis with water (20 mL) and extraction with diethyl ether (3 x 20 mL), the solutions were combined, dried with $MgSO_4$ and concentrated to dryness in vacuo. The residue was recrystallized from diethyl ether to afford **1b** (11.2 g, 85%) as colorless crystals. M.P.: 154-156°C. ^{1}H NMR (CDCl$_3$): δ 0.33 (s, 12H, Si(C*H*$_3$)$_2$), 7.45 (m, 4H, SiC$_6$*H*$_5$), 7.50 (m, 2H, SiC$_6$*H*$_5$) , 7.54 (m, 4H, SiC$_6$*H*$_5$). ^{13}C{^{1}H} NMR (CDCl$_3$): δ 0.1 (Si(*C*H$_3$)$_2$), 74.8 (cage *C*), 127.9, 130.7, 134.0, 135.4 (Si*C*$_6$H$_5$). ^{11}B{^{1}H} NMR (CDCl$_3$): δ 1.4 (2B), -6.9 (2B), -9.6 (4B), -11.5 (2B). HRMS: *m/z* calcd for $C_{17}H_{29}B_{10}Si_2$ [M-CH$_3$]$^+$: 398.2786. Found: 398.2784.

Preparation of 1,12-TMS$_2$-1,12-C$_2$B$_{11}$H$_{11}$ (2a). To a THF (30 mL) solution of **1a** (2.89 g, 10.0 mmol) was added finely cut Na metal (0.69 g, 30.0 mmol), and the mixture was stirred at room temperature for 1 day. Removal of excess Na metal and THF afforded a pale yellow solid. DME (20 mL) was then added, giving a yellow solution. HBBr$_2$·SMe$_2$ (20.0 mL of 1.0 M in dichloromethane, 20.0 mmol) was slowly added to the solution at -78°C, the mixture was stirred at this temperature for 1 h, and then at room temperature overnight. After removal of the solvent, the residue was subjected to chromatographic separation (SiO$_2$, 300 ~ 400 mesh, *n*-hexane as eluent), affording **1a** (0.35 g, 12%) and **2a** (0.60 g, 20%) both as white solid. X-ray-quality crystals of **2a** were obtained by recrystallization from *n*-hexane. M.P.: 40-41°C. ^{1}H NMR (CDCl$_3$): δ 0.32 (s, 9H, Si(C*H*$_3$)$_3$), 0.20 (s, 9H, Si(C*H*$_3$)$_3$). ^{13}C{^{1}H} NMR (CDCl$_3$): δ 120.5 (cage *C*), 98.4 (cage *C*), -1.0, -1.2 (Si(*C*H$_3$)$_3$). ^{11}B{^{1}H} NMR (CDCl$_3$): δ 15.2 (1B), 8.5 (2B), 0.0 (1B), -2.6 (3B), -7.5 (4B). HRMS: *m/z* calcd for $C_8H_{27}B_{11}Si_2$ [M – 2H]$^+$: 298.2750. Found: 298.2747.

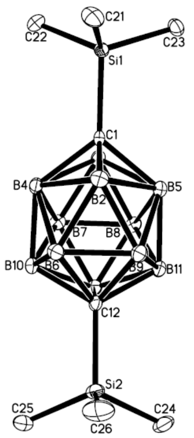

Supplementary Figure 1. Molecular structure of **2a** (hydrogen atoms are omitted for clarity).

Preparation of 1,12-DMPS$_2$-1,12-$C_2B_{11}H_{11}$ (2b). To a THF (40 mL) solution of **1b** (8.23 g, 20.0 mmol) was added finely cut Na metal (0.93 g, 40.6 mmol), and the mixture was stirred at room temperature for 1 day. Removal of excess Na metal and THF afforded a yellow solid. DME (30 mL) was then added, giving a yellow solution. $HBBr_2{\cdot}SMe_2$ (40.0 mL of 1.0 M in dichloromethane, 40.0 mmol) was slowly added to the solution at -78°C, and the mixture was stirred at this temperature for 1 h, and then at room temperature overnight. After removal of the solvent, the residue was subjected to chromatographic separation (SiO_2, 300 ~ 400 mesh) using *n*-hexane/CH_2Cl_2 (4/1 in v/v) as eluent to give **1b** (0.36 g, 4%) and **2b** (2.48 g, 29%) both as white solid. X-ray-quality crystals of **2b** were obtained by recrystallization from *n*-hexane. M.P.: 89-90°C. ^{1}H NMR ($CDCl_3$): δ 0.52 (s, 12H, Si(CH_3)$_2$), 7.38 (m, 5H, SiC_6H_5), 7.47 (m, 5H, SiC_6H_5). ^{13}C{^{1}H} NMR ($CDCl_3$): δ -2.9 (Si(CH_3)$_2$), 97.6 (cage *C*), 119.6 (cage *C*), 127.9, 128.0, 130.1, 130.2, 134.4, 134.5 (SiC_6H_5). ^{11}B{^{1}H} NMR ($CDCl_3$): δ 14.7 (1B), 8.1 (2B), -0.7 (1B), -2.8 (3B), -8.1 (3B), -16.8 (1B). HRMS: *m/z* calcd for $C_{18}H_{33}B_{11}Si_2$ $[M]^+$: 423.3146. Found: 423.3144.

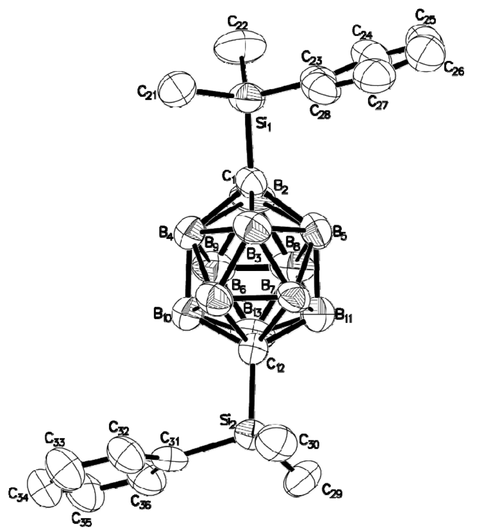

Supplementary Figure 2. Molecular structure of **2b** (hydrogen atoms are omitted for clarity).

Preparation of 2,9-TMS$_2$-2,9-C$_2$B$_{12}$H$_{12}$ (3a). To a THF (30 mL) solution of **2a** (3.00 g, 10.0 mmol) was added finely cut Na metal (0.69 g, 30.0 mmol), and the mixture was stirred at room temperature overnight. Removal of excess Na metal and THF afforded a pale yellow solid. CH_2Cl_2 (20 mL) was then added, giving a yellow solution. $HBBr_2 \cdot SMe_2$ (30.0 mL of 1.0 M in dichloromethane, 30.0 mmol) was slowly added to the solution at -78°C, the mixture was stirred at this temperature for 1 h, and then at room temperature overnight. After removal of the solvent, the residue was subjected to chromatographic separation (SiO_2, 300 ~ 400 mesh, *n*-hexane as eluent), affording **2a** (0.06 g, 2%) and **3a** (0.62 g, 20%) both as white solid. X-ray-quality crystals of **3a** were obtained by recrystallization from *n*-hexane. M.P.: 50-51°C. ^{1}H NMR ($CDCl_3$): δ 0.27 (s, 18H, Si(C*H*$_3$)$_3$). ^{13}C{^{1}H} NMR ($CDCl_3$): δ 82.5 (cage *C*), -1.6 (Si(*C*H$_3$)$_3$). ^{11}B{^{1}H} NMR ($CDCl_3$): δ 9.9 (2B), -3.6 (4B), -10.0 (4B), -20.6 (2B). HRMS: *m/z* calcd for $C_8H_{30}B_{12}Si_2$ [M]$^+$: 312.3078. Found: 312.3066.

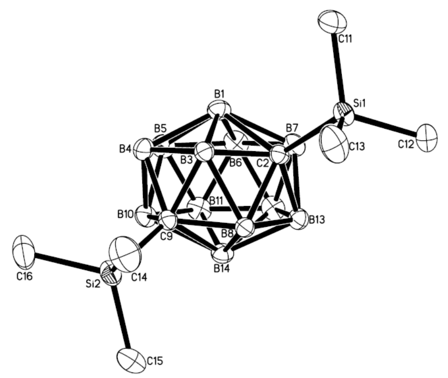

Supplementary Figure 3. Molecular structure of **3a** (hydrogen atoms are omitted for clarity).

Preparation of 2,9-DMPS$_2$-2,9-C$_2$B$_{12}$H$_{12}$ (3b). To a THF (20 mL) solution of **2b** (3.20 g, 7.59 mmol) was added finely cut Na metal (0.89 g, 38.6 mmol), and the mixture was stirred at room temperature overnight. Removal of excess Na metal and THF afforded a pale yellow solid. CH_2Cl_2 (25 mL) was then added, giving a yellow solution. $HBBr_2 \cdot SMe_2$ (25.0 mL of 1.0 M in dichloromethane, 25.0 mmol) was slowly added to the solution at -78°C, the mixture was stirred at this temperature for 1 h, and then at room temperature overnight. After removal of the solvent, the residue was subjected to chromatographic separation (SiO_2, 300 ~ 400 mesh) using *n*-hexane/CH_2Cl_2 (4/1 in v/v) as eluent to give **2b** (0.10 g, 3%) and **3b** (0.49 g, 15%) both as white solid. X-ray-quality crystals of **3b** were obtained by recrystallization from *n*-hexane. M.P.: 123-125°C. ^{1}H NMR ($CDCl_3$): δ 0.39 (s, 6H, Si(CH_3)$_2$), 0.47 (s, 6H, Si(CH_3)$_2$), 7.37 (m, 4H, SiC$_6$$H_5$), 7.43 (m, 2H, SiC$_6$$H_5$), 7.49 (m, 4H, SiC$_6$$H_5$). ^{13}C{^{1}H} NMR ($CDCl_3$): δ -3.5 (Si(CH$_3$)$_2$), 82.0 (cage *C*), 127.9, 130.4, 133.7, 134.8 (SiC_6H$_5$). ^{11}B{^{1}H} NMR ($CDCl_3$): δ 9.6 (2B), -3.9 (4B), -10.2 (4B), -20.5 (2B). HRMS: *m/z* calcd for $C_{18}H_{34}B_{12}Si_2$ [M]$^+$: 436.3397. Found: 436.3399.

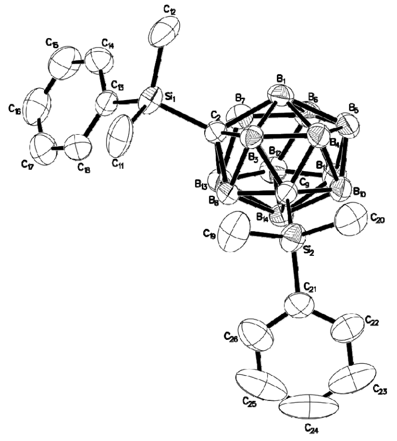

Supplementary Figure 4. Molecular structure of **3b** (hydrogen atoms are omitted for clarity).

Preparation of 1,14-TMS$_2$-1,14-C$_2$B$_{13}$H$_{13}$ (4a). To a THF (30 mL) solution of **3a** (3.12 g, 10.0 mmol) was added finely cut Na metal (0.69 g, 30.0 mmol), and the mixture was stirred at room temperature overnight. Removal of excess Na metal and THF afforded a pale yellow solid. CH_2Cl_2 (20 mL) was then added, giving a yellow solution. $HBBr_2 \cdot SMe_2$ (30.0 mL of 1.0 M in dichloromethane, 30.0 mmol) was slowly added to the solution at -78°C, the mixture was stirred at this temperature for 1 h, and then at room temperature overnight. After removal of the solvent, the residue was subjected to chromatographic separation (SiO_2, 300 ~ 400 mesh, *n*-hexane as eluent), affording **3a** (0.16 g, 5%) and **4a** (0.26 g, 8%) both as white solid. ^{1}H NMR ($CDCl_3$): δ 0.42 (s, 18H, Si(CH_3)$_3$). ^{13}C{^{1}H} NMR ($CDCl_3$): δ -0.3 (Si(CH$_3$)$_3$), the

cage *C* atoms were not observed. ^{11}B{^{1}H} NMR ($CDCl_3$): δ 16.3 (2B), -1.0 (4B), -4.5 (4B), -6.1 (1B), -20.5 (2B). HRMS: *m/z* calcd for $C_8H_{31}B_{13}Si_2$ $[M]^+$: 324.3251. Found: 324.3244.

Preparation of 1,14-DMPS$_2$-1,14-$C_2B_{13}H_{13}$ (4b). To a THF (15 mL) solution of **3b** (640 mg, 1.47 mmol) was added finely cut Na metal (98 mg, 4.26 mmol), and the mixture was stirred at room temperature overnight. Removal of excess Na metal and THF afforded a pale yellow solid. CH_2Cl_2 (4.5 mL) was then added, giving a pale yellow solution. $HBBr_2{\cdot}SMe_2$ (4.50 mL of 1.0 M in dichloromethane, 4.50 mmol) was slowly added to the solution at -78°C, the mixture was stirred at this temperature for 1 h, and then at room temperature overnight. After removal of the solvent, the residue was subjected to chromatographic separation (SiO_2, 300 ~ 400 mesh) using *n*-hexane/CH_2Cl_2 (4/1 in v/v) as eluent to give **3b** (36 mg, 6%) and **4b** (23 mg, 3%) both as white solid. X-ray-quality crystals of **4b** were obtained by recrystallization from *n*-hexane. M.P.: 133-134°C. ^{1}H NMR ($CDCl_3$): δ 0.70 (s, 12H, Si(CH_3)$_2$), 7.41 (m, 6H, SiC_6H_5), 7.63 (m, 4H, SiC_6H_5). ^{13}C{^{1}H} NMR ($CDCl_3$): δ -2.1 (Si(CH_3)$_2$), 57.7 (cage *C*), 127.9, 130.2, 134.5, 135.0 (SiC_6H_5). ^{11}B{^{1}H} NMR ($CDCl_3$): δ 15.0 (2B), -1.8 (4B), -5.7 (4B), -10.8 (1B), -21.3 (2B). HRMS: *m/z* calcd for $C_{18}H_{35}B_{13}Si_2$ $[M]^+$: 448.3554. Found: 448.3558.

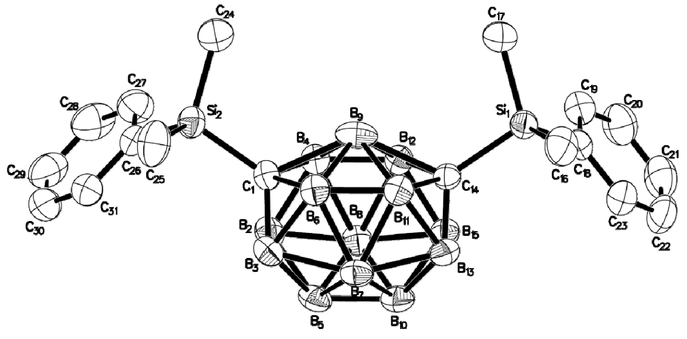

Supplementary Figure 5. Molecular structure of **4b** (hydrogen atoms are omitted for clarity).

Preparation of 9,11-TMS$_2$-1-(*p*-cymene)-1,9,11-Ru$C_2B_{13}H_{13}$ (5Ru). To a THF (10 mL) solution of **4a** (324 mg, 1.0 mmol) was added finely cut Na metal (69 mg, 3.0 mmol), and the mixture was stirred at room temperature for one day. Removal of excess Na metal gave a clear yellow solution. The above solution was slowly added to a THF (10 mL) suspension of [(*p*-cymene)RuCl_2]$_2$ (306 mg, 0.50 mmol) at -30°C, the mixture was then stirred at room temperature overnight to give a deep brown solution. Removal of the precipitate and solvent gave a deep brownish sticky solid. Chromatographic separation (SiO_2, 300-400 mesh, using *n*-hexane/CH_2Cl_2 (4:1 in V/V as eluent) gave **4a** (81 mg, 25%) as a white solid and **5Ru** as a

yellow solid (213 mg, 38%). X-ray-quality crystals of **5Ru** were obtained by recrystallization from toluene. M.P.: 130-131°C. ^{1}H NMR (CD_2Cl_2): δ 5.65 (d, J = 6.3 Hz, 2H, C_6H_4), 5.56 (d, J = 6.3 Hz, 2H, C_6H_4), 2.81 (m, 1H, C_6H_4CH), 2.15 (s, 3H, $C_6H_4CH_3$), 1.23 (d, J = 6.9 Hz, 6H, $CHCH_3$), 0.25 (s, 18H, $SiCH_3$). ^{13}C{^{1}H} NMR (CD_2Cl_2): δ 118.9, 109.2, 97.3, 94.4 (C_6H_4), 55.5 (cage *C*), 29.9 ($C_6H_4CH_3$), 22.7 ($CHCH_3$), 18.0 (C_6H_4CH), 0.2 ($Si(CH_3)_3$). ^{11}B{^{1}H} NMR CD_2Cl_2): δ 0.1 (2B), -10.3 (2B), -13.0 (2B), -16.8 (1B), -21.3 (4B), -28.2 (2B). HRMS: *m/z* calcd for $C_{18}H_{45}B_{13}RuSi_2$ $[M]^+$: 559.3411; Found: 559.3420.

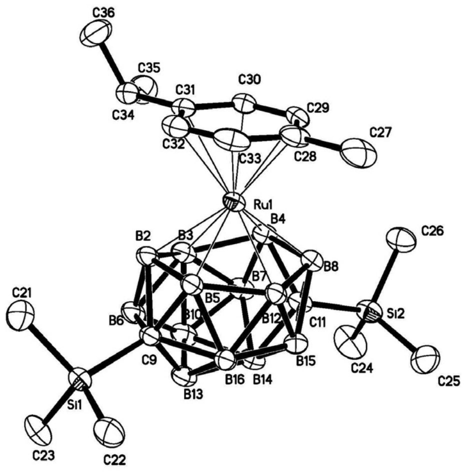

Supplementary Figure 6. Molecular structure of **5Ru** (hydrogen atoms are omitted for clarity).

Preparation of 5,6-(DMPS)$_2$-5,6-$C_2B_{14}H_{14}$ (5b). To a THF (5 mL) solution of **4b** (53 mg, 0.1 mmol) was added finely cut Na metal (7 mg, 0.3 mmol), and the mixture was stirred at room temperature for 3 days. Removal of excess Na metal and THF afforded a colorless solid. CH_2Cl_2 (0.4 mL) was then added, giving a yellow solution. $HBBr_2{\cdot}SMe_2$ (0.4 mL of 1.0 M in dichloromethane, 0.4 mmol) was slowly added to the solution at -78°C, the mixture was stirred at this temperature for 1 h, and then at room temperature overnight. After hydrolysis with water and extraction with dichloromethane (5 mL x 3), the solutions were combined, dried with $MgSO_4$ and concentrated to dryness in vacuo. Chromatographic separation (SiO_2, 300 ~ 400 mesh, *n*-hexane/CH_2Cl_2 (4/1 in v/v) as eluent) afforded **5b** (14 mg, 26%) as a white solid. X-ray-quality crystals of **5b** were obtained by recrystallization from *n*-hexane. M.P.: 148-149°C. ^{1}H NMR (400 MHz, $CDCl_3$): δ 0.46 (s, 6H, $Si(CH_3)_2$), 0.59 (s, 6H, $Si(CH_3)_2$), 7.48, (m, 6H, SiC_6H_5), 7.64 (d, 4H, SiC_6H_5). ^{13}C{^{1}H} NMR (125 MHz, $CDCl_3$): δ -2.7

(Si(CH_3)$_2$), 59.4 (cage *C*), 128.1, 130.8, 134.1, 135.2 (SiC_6H_5). $^{11}B\{^1H\}$ NMR (128 MHz, $CDCl_3$): δ 14.3 (1B), -0.9 (4B), -13.0 (4B), -16.5 (3B), -18.3 (2B). HRMS: *m/z* calcd for $C_{18}H_{36}B_{14}Si_2$ $[M]^+$: 460.3742. Found: 460.3749.

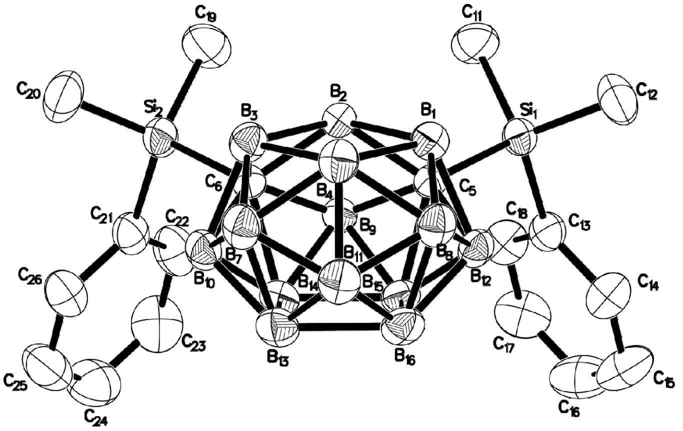

Supplementary Figure 7. Molecular structure of **5b** (hydrogen atoms are omitted for clarity).

X-ray Structure Determination. All data were collected at 293 K on a Bruker SMART 1000 CCD diffractometer using Mo-Kα radiation. An empirical absorption correction was applied using the SADABS program.[2] All structures were solved by direct methods and subsequent Fourier difference techniques and refined anisotropically for all non-hydrogen atoms by full-matrix least squares calculations on F^2 using the SHELXTL program package.[3] All hydrogen atoms were geometrically fixed using the riding model.

Details of the crystal structures were deposited in the Cambridge Crystallographic Data Centre with CCDC 1997899-1997905 for **2a**, **2b**, **3a**, **3b**, **4b**, **5b**, and **5Ru**, respectively.

Computational detail

All of these calculations were carried out with the Gaussian 09 program.[4] Optimization of compounds **4b** and **5b** were performed at B3LYP-D3/6-31G(d,p) level of theory.[5] Frequency calculations were made to determine the characteristics of all stationary points as energy minima. The orbital energies were calculated at the same level. The graphics of molecular orbitals were produced by using the visualizing software VMD.[6]

For Cartesian coordinates in .txt format for the optimized structures of **4b** and **5b**, see: Supplementary Data 1 and Supplementary Data 2.

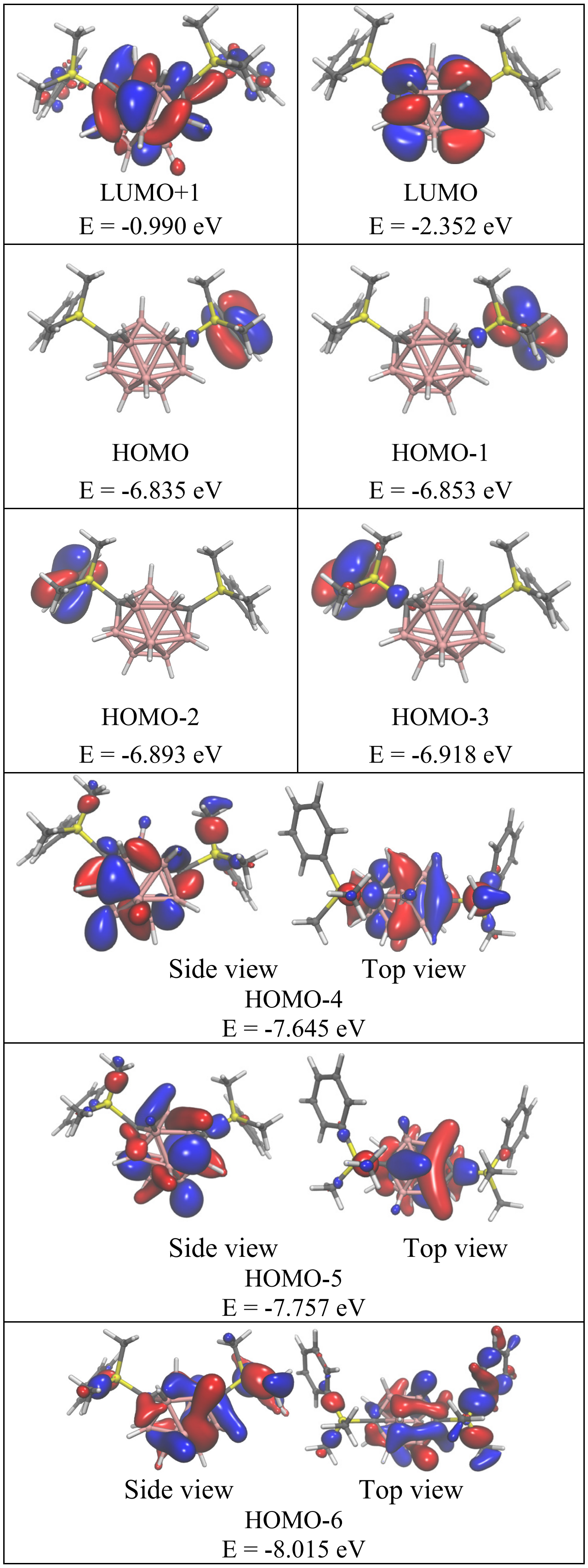

Supplementary Figure 8. Selected calculated molecular orbitals of 4b

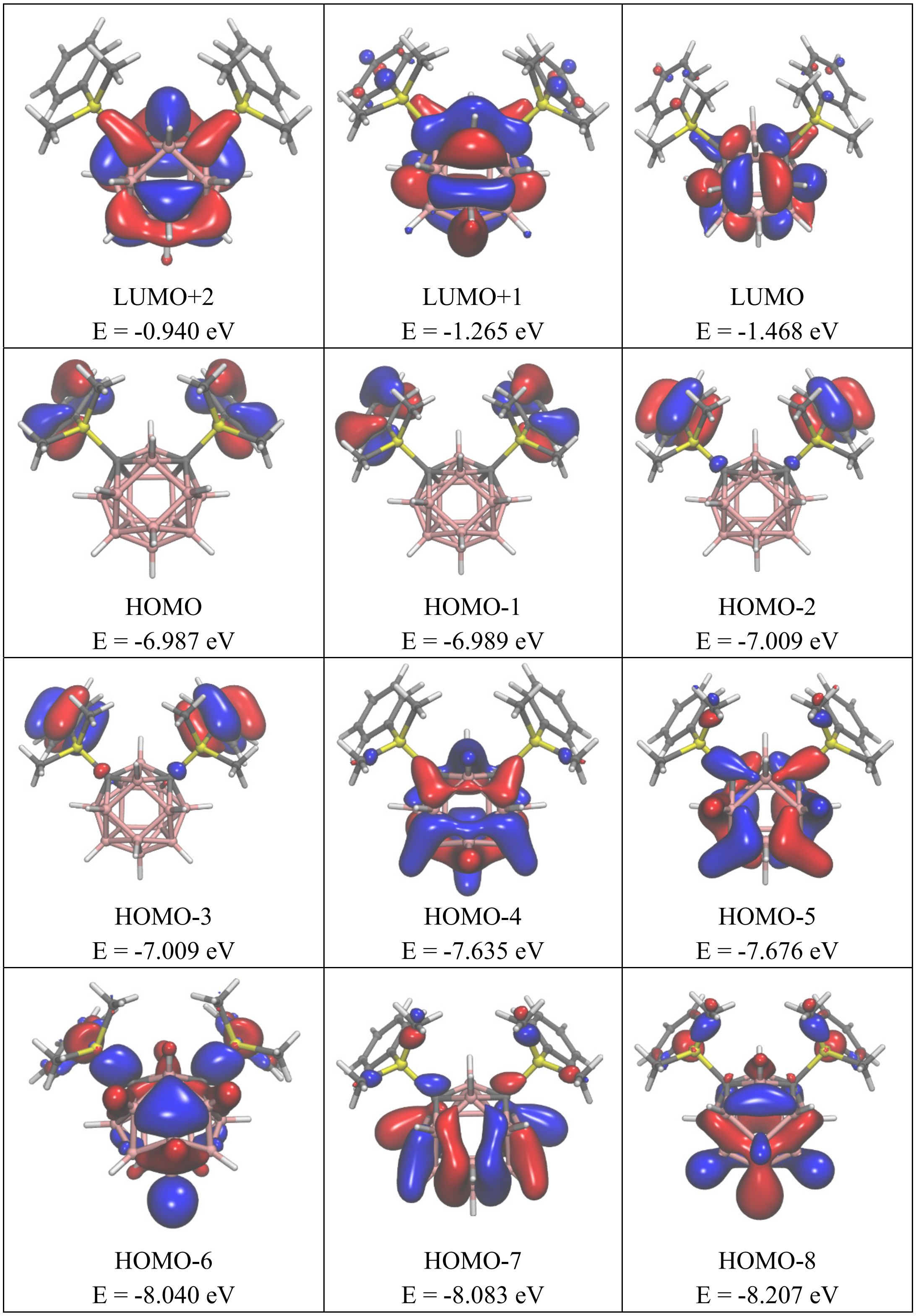

Supplementary Figure 9. Selected calculated molecular orbitals of 5b

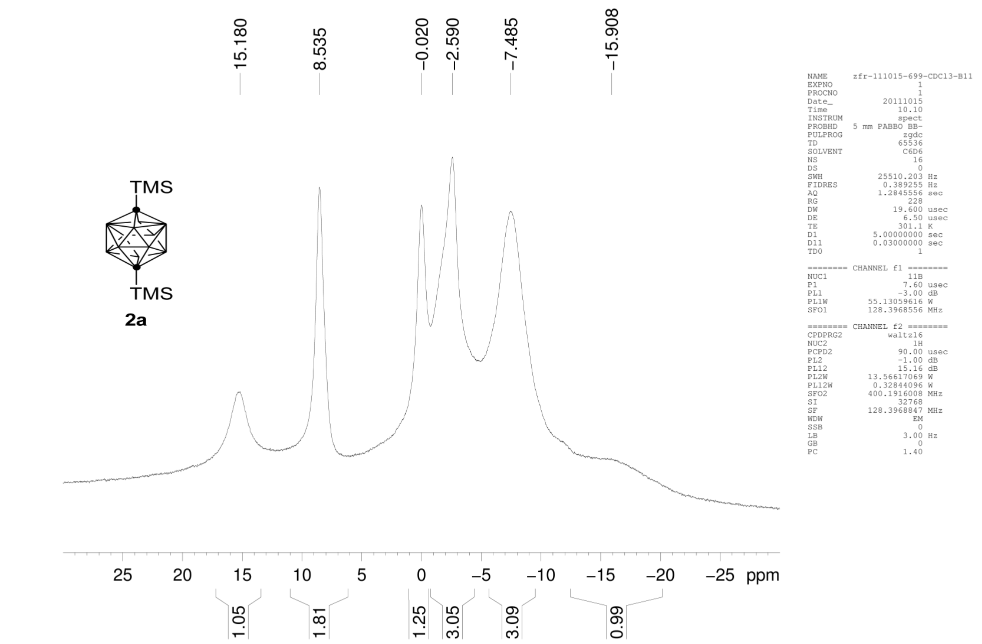

Supplementary Figure 10. $^{11}B\{^{1}H\}$ NMR spectrum of **2a** in $CDCl_3$.

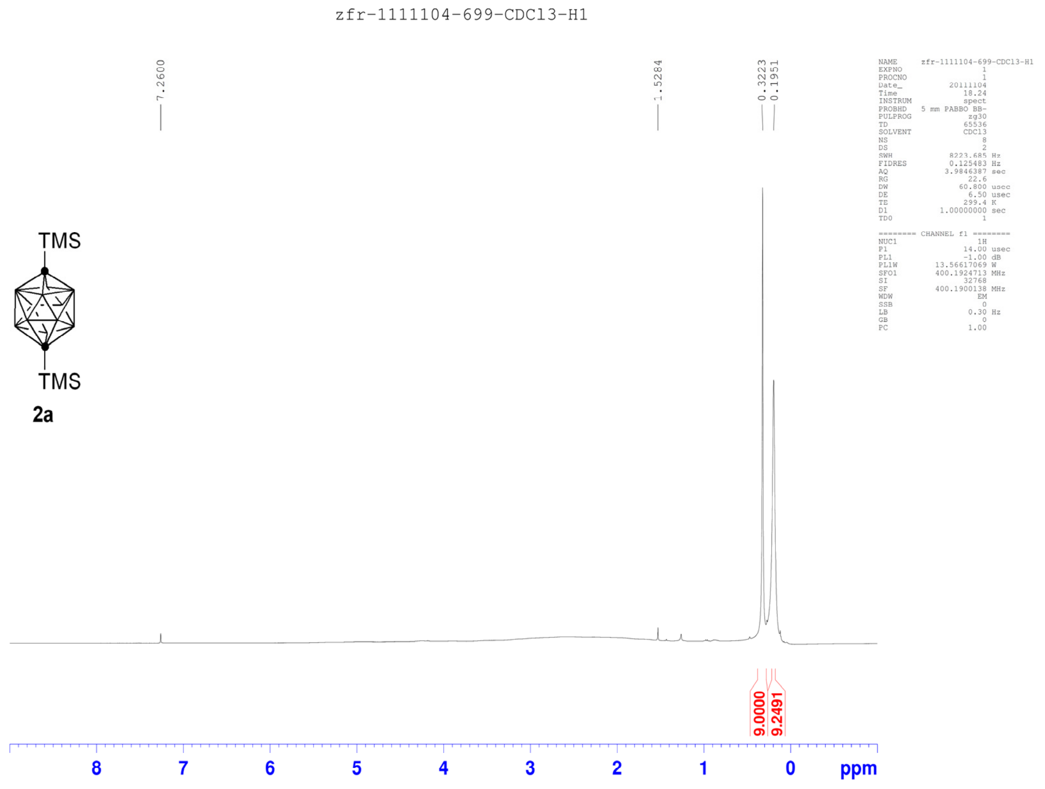

Supplementary Figure 11. ^{1}H NMR spectrum of **2a** in $CDCl_3$.

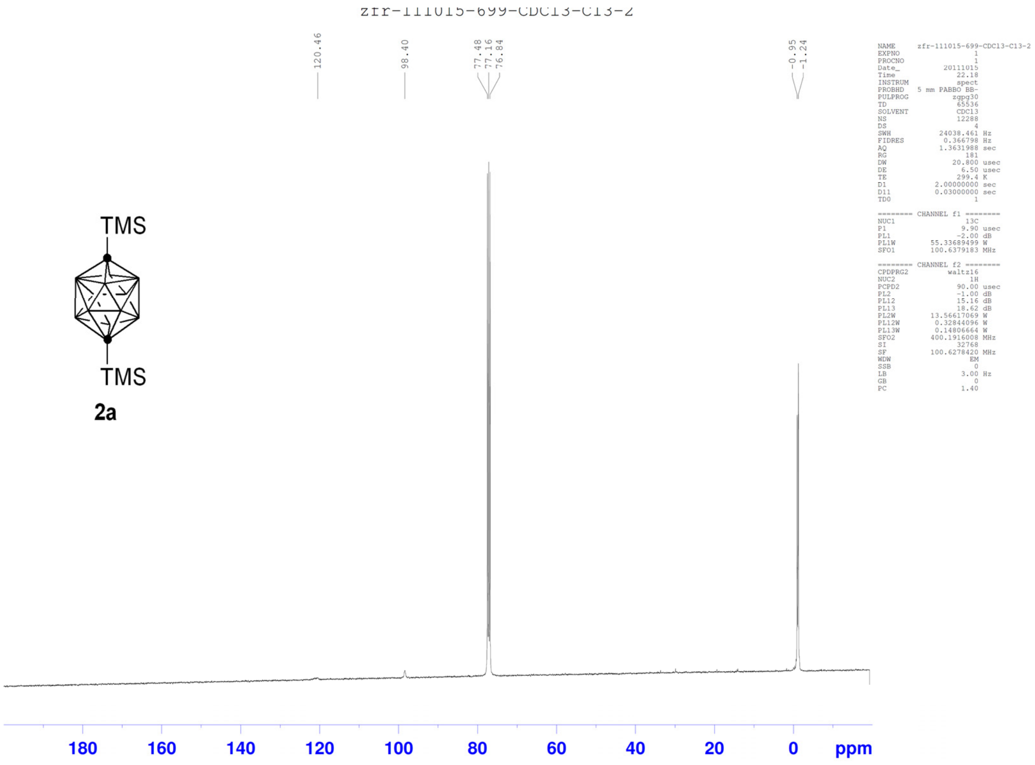

Supplementary Figure 12. ^{13}C NMR spectrum of **2a** in $CDCl_3$.

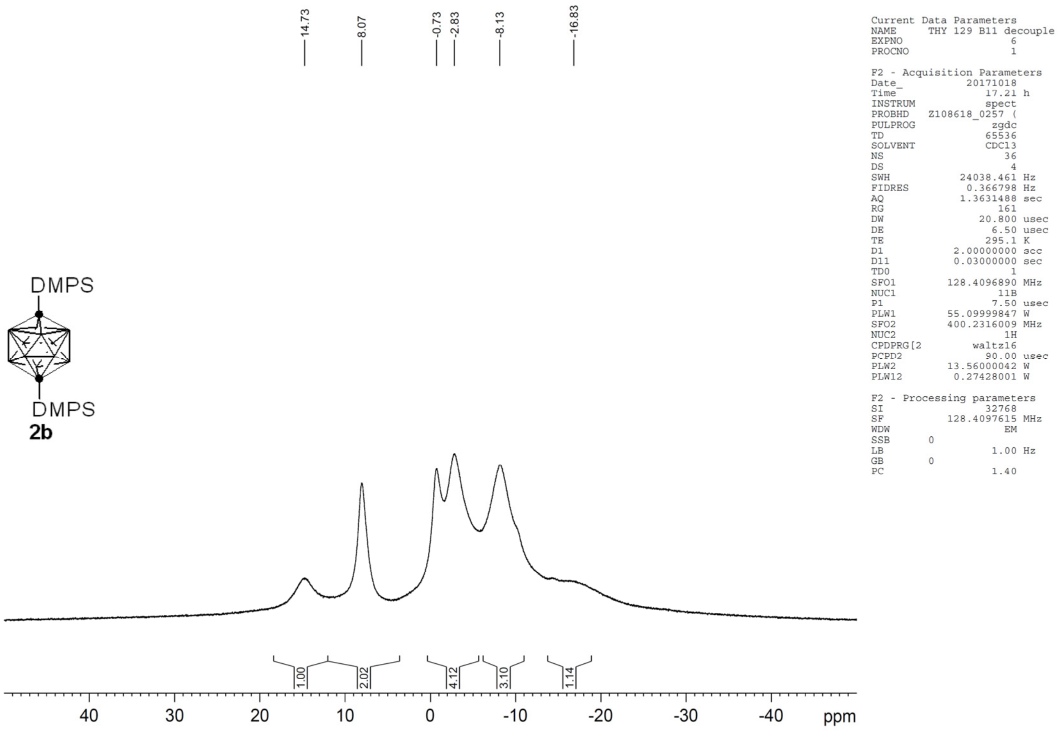

Supplementary Figure 13. $^{11}B\{^{1}H\}$ NMR spectrum of **2b** in $CDCl_3$.

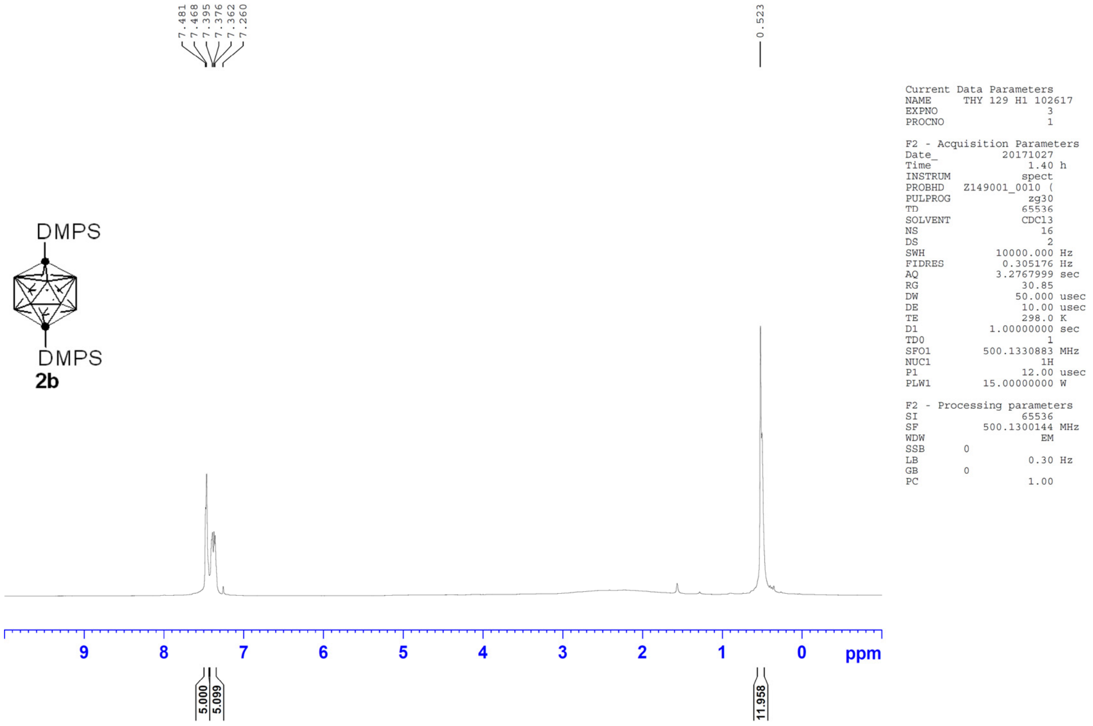

Supplementary Figure 14. ^{1}H NMR spectrum of **2b** in $CDCl_3$.

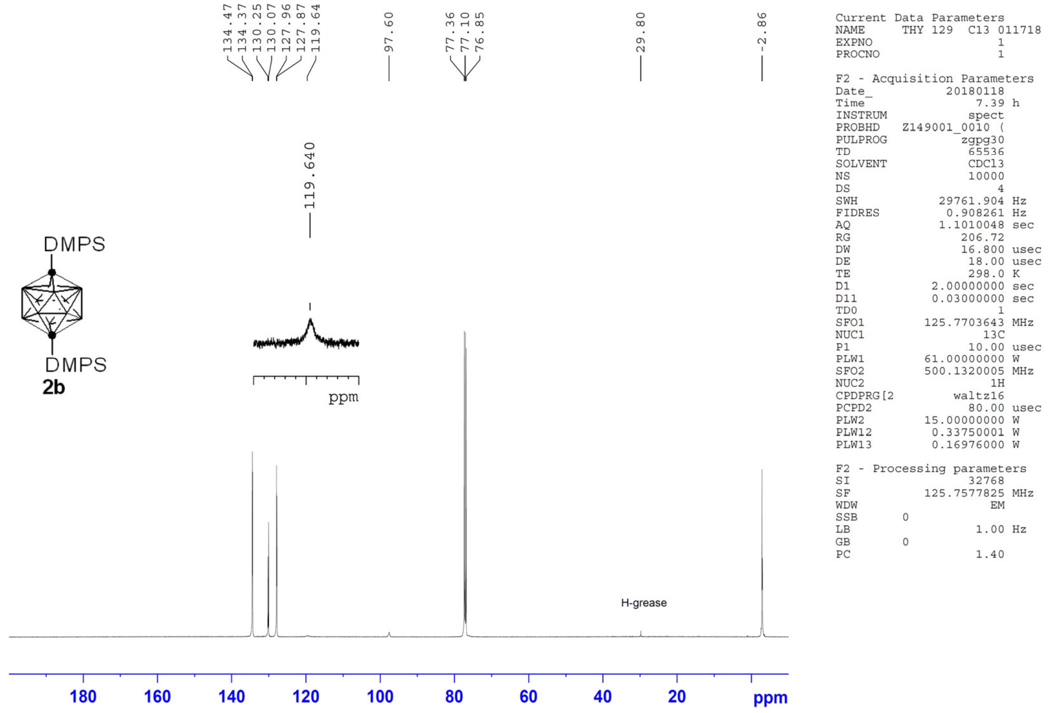

Supplementary Figure 15. ^{13}C NMR spectrum of **2b** in $CDCl_3$.

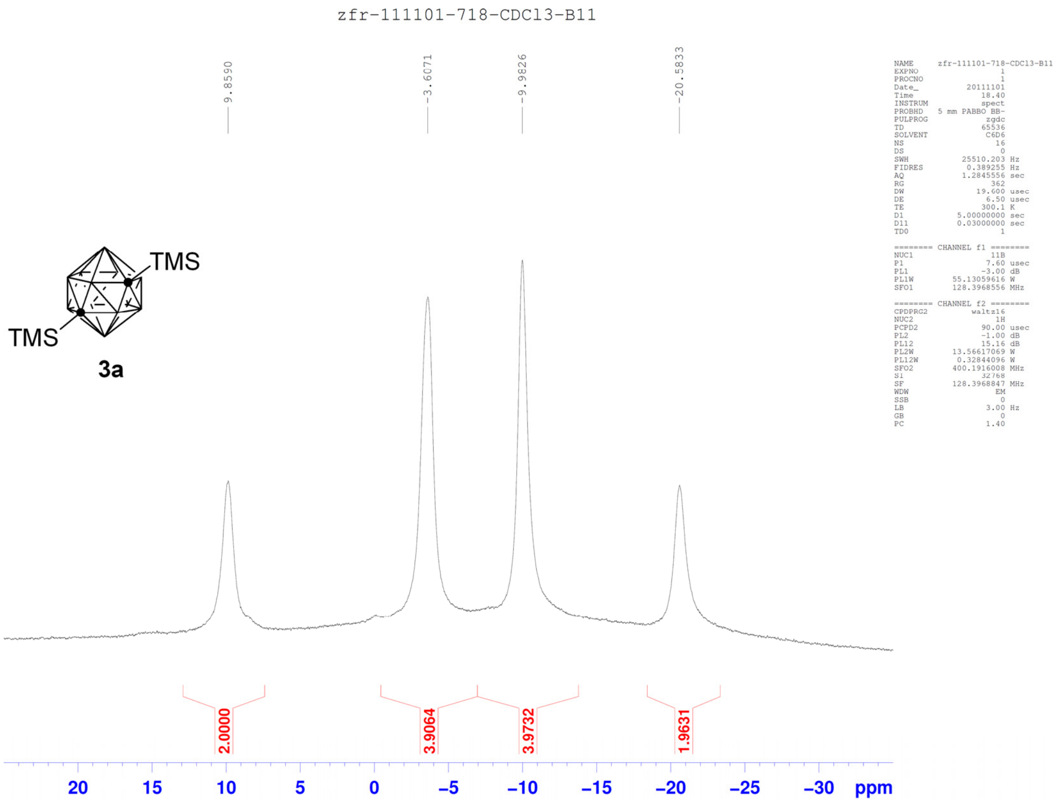

Supplementary Figure 16. $^{11}B\{^{1}H\}$ NMR spectrum of **3a** in $CDCl_3$.

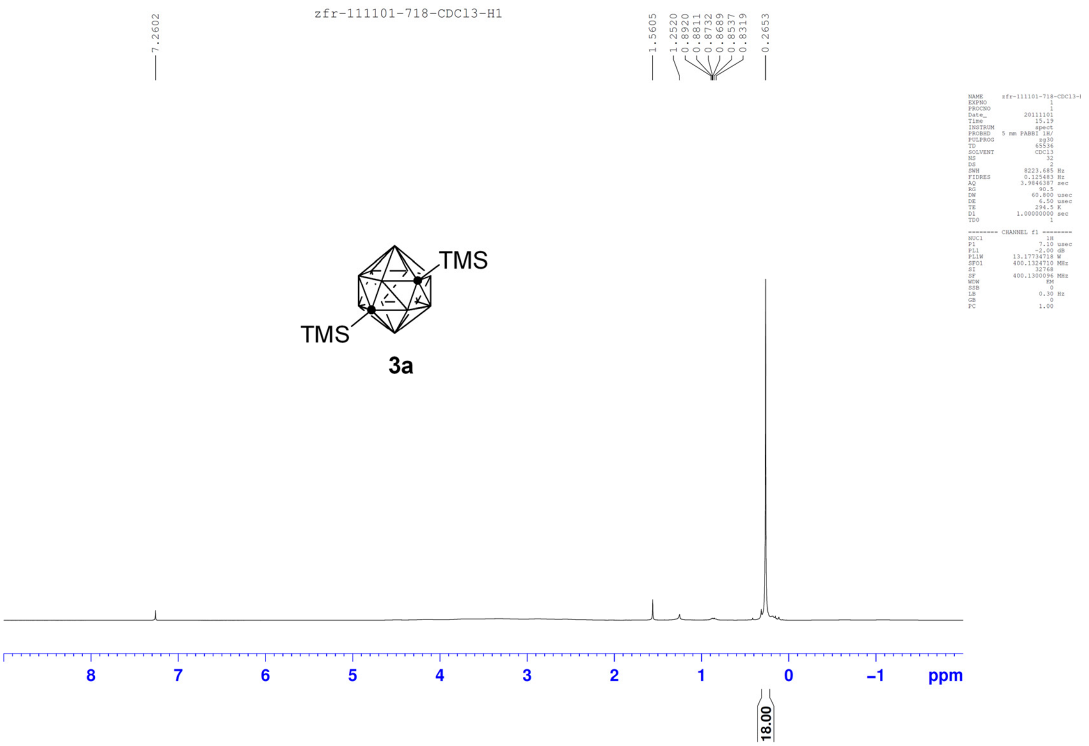

Supplementary Figure 17. ^{1}H NMR spectrum of **3a** in $CDCl_3$.

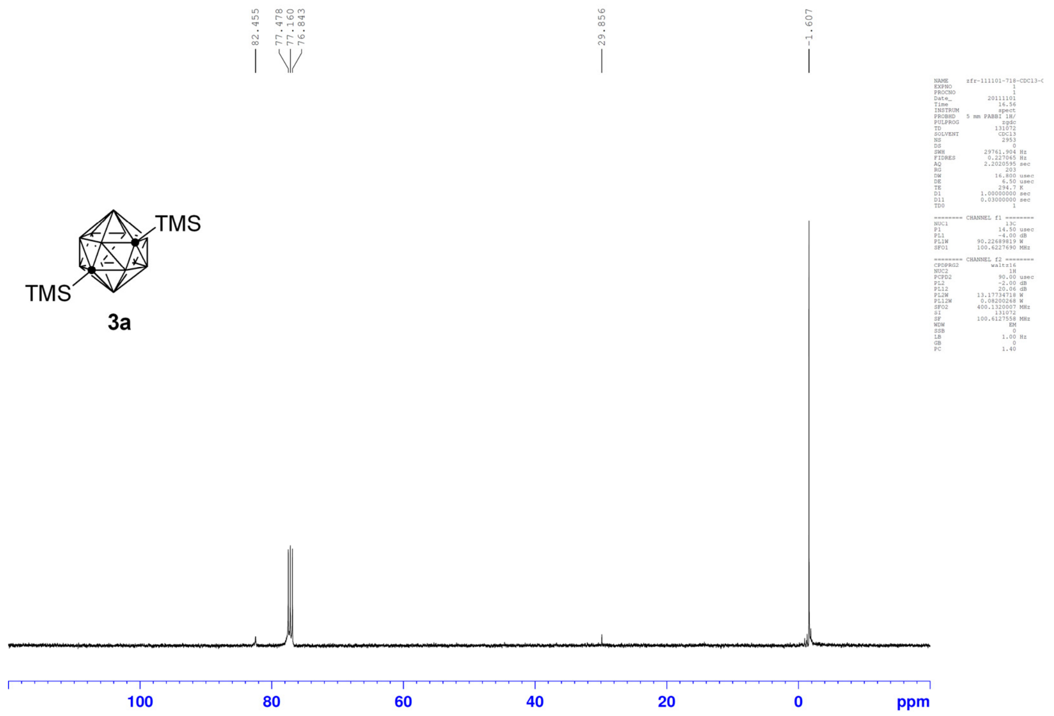

Supplementary Figure 18. ^{13}C NMR spectrum of **3a** in $CDCl_3$.

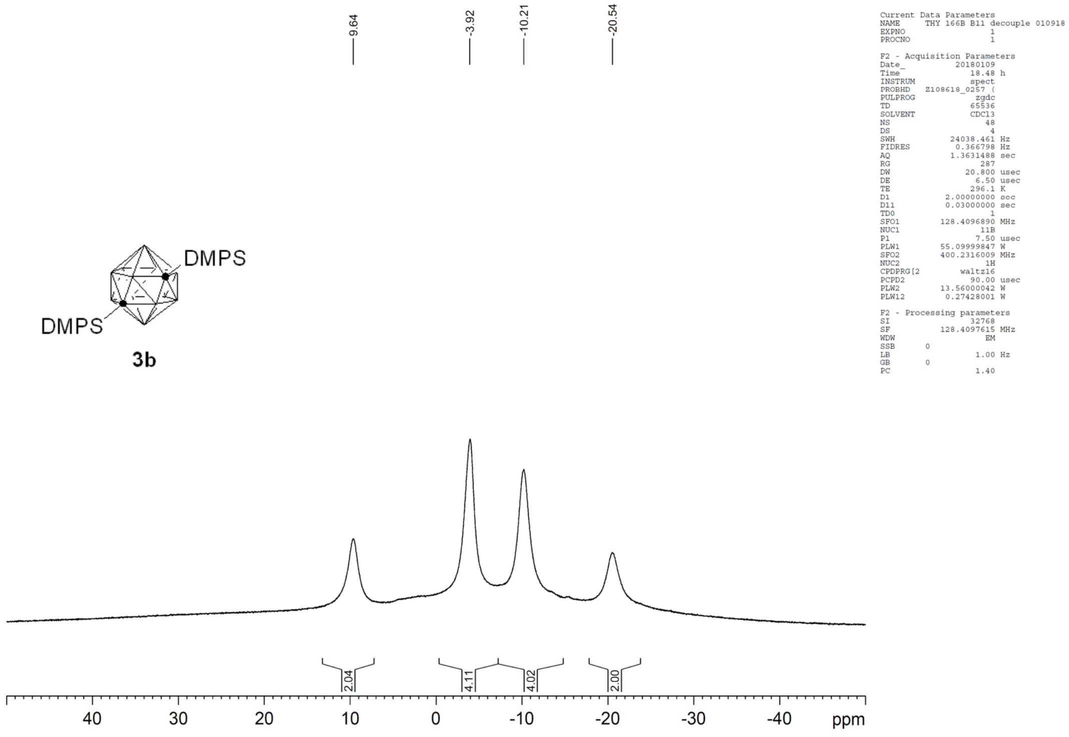

Supplementary Figure 19. $^{11}B\{^{1}H\}$ NMR spectrum of **3b** in $CDCl_3$.

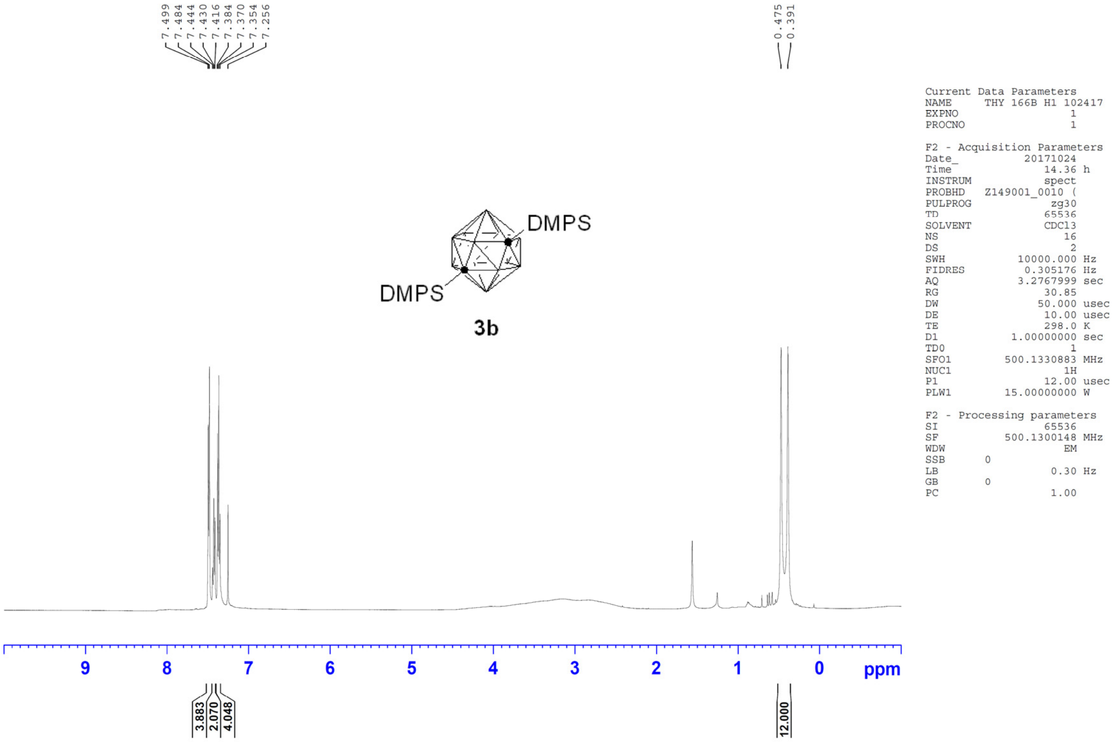

Supplementary Figure 20. ^{1}H NMR spectrum of **3b** in $CDCl_3$.

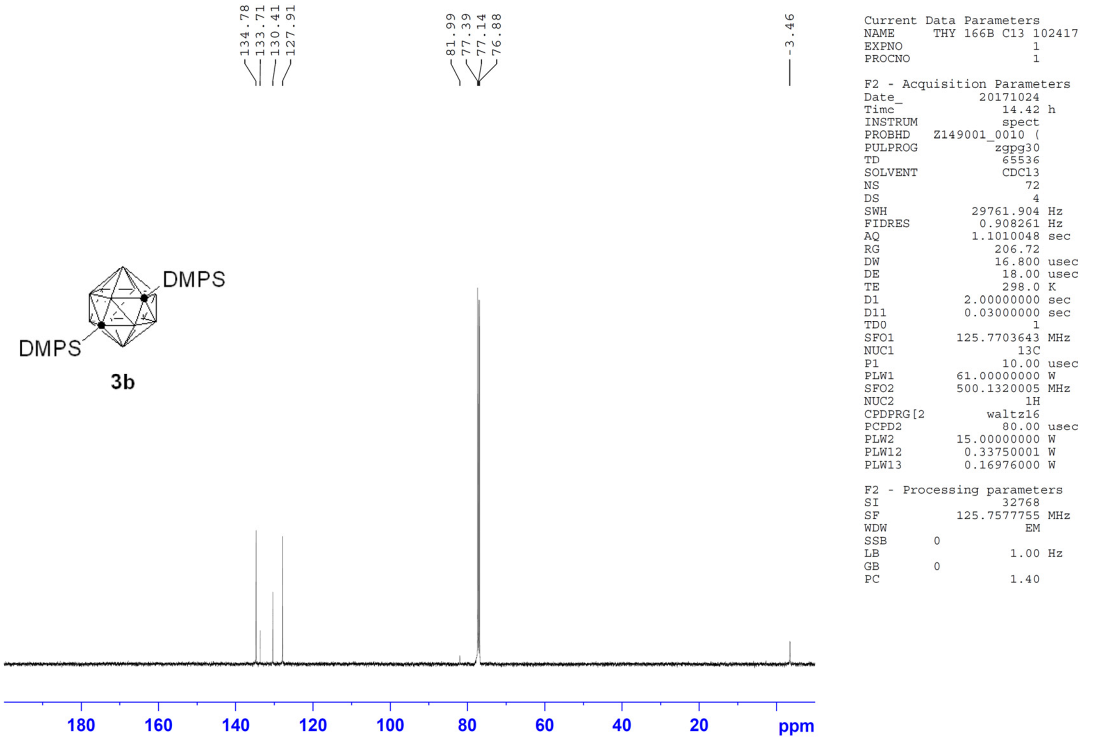

Supplementary Figure 21. ^{13}C NMR spectrum of **3b** in $CDCl_3$.

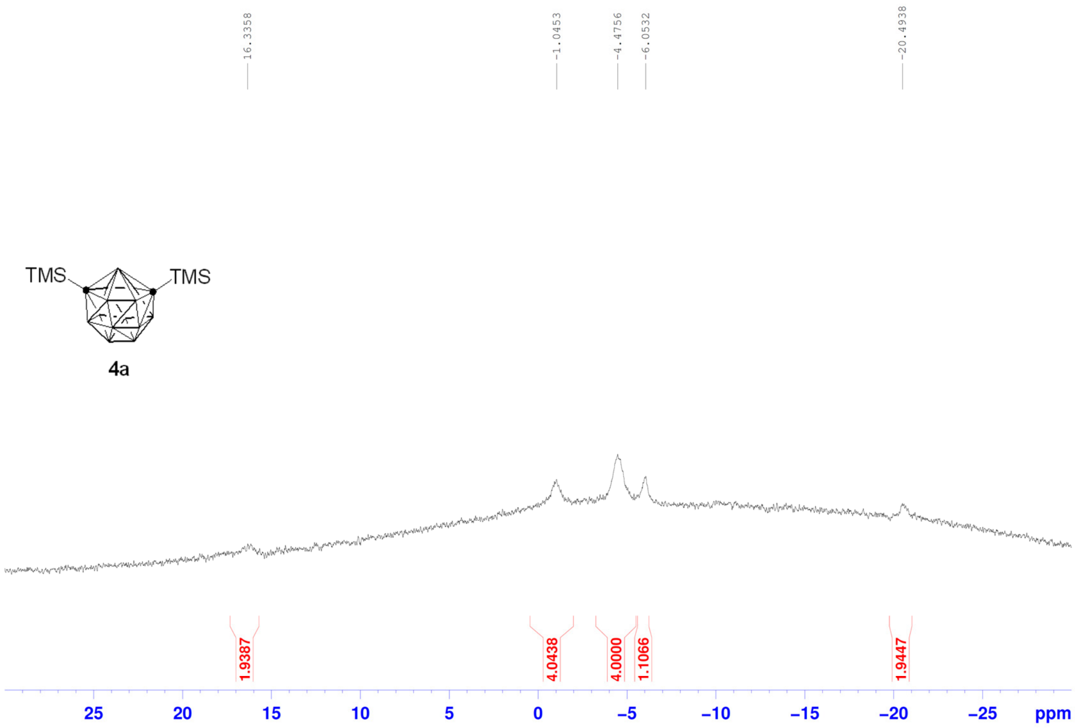

Supplementary Figure 22. $^{11}B\{^{1}H\}$ NMR spectrum of **4a** in $CDCl_3$.

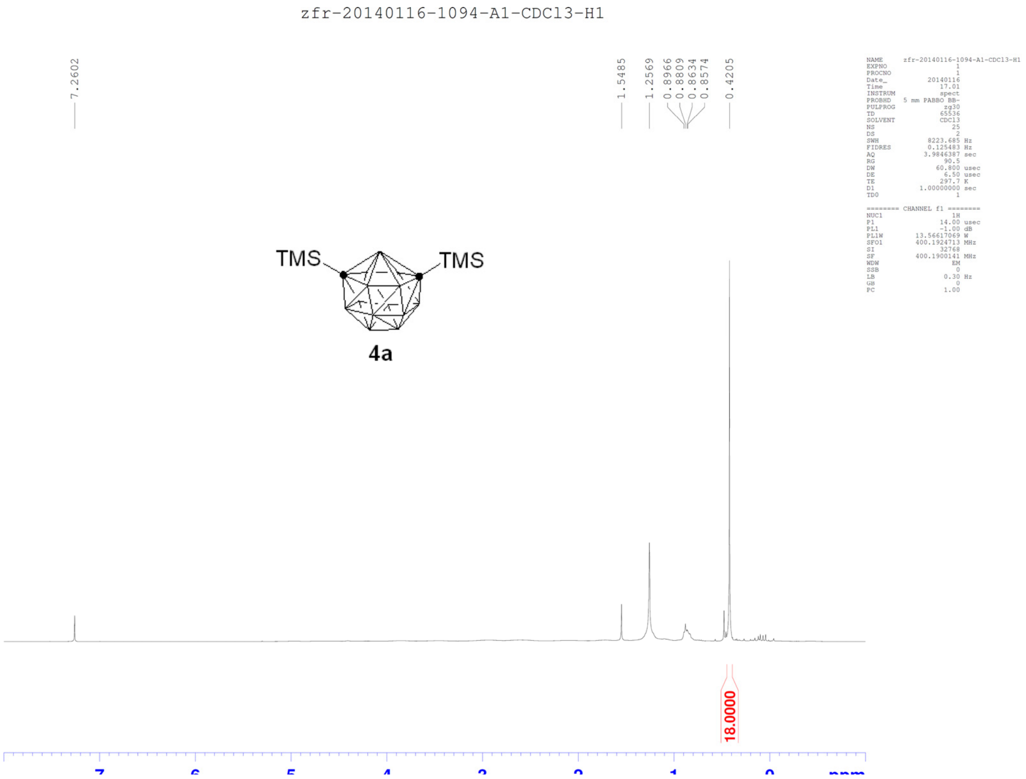

Supplementary Figure 23. ^{1}H NMR spectrum of **4a** in $CDCl_3$.

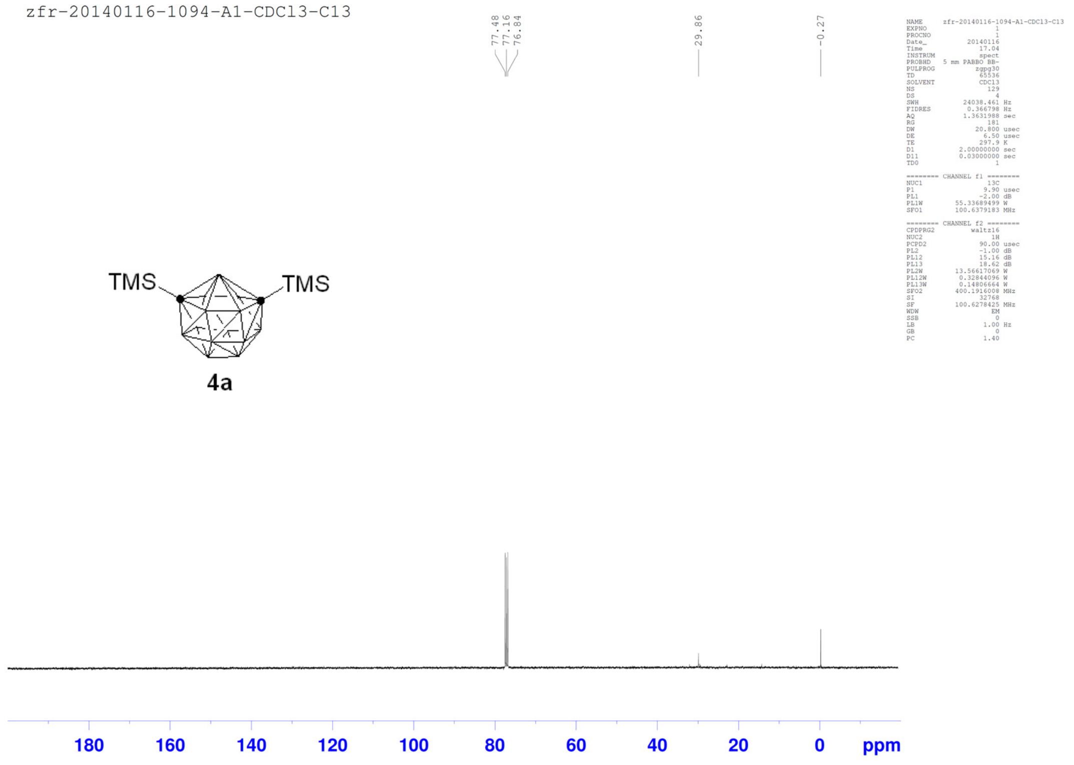

Supplementary Figure 24. ^{13}C NMR spectrum of **4a** in $CDCl_3$.

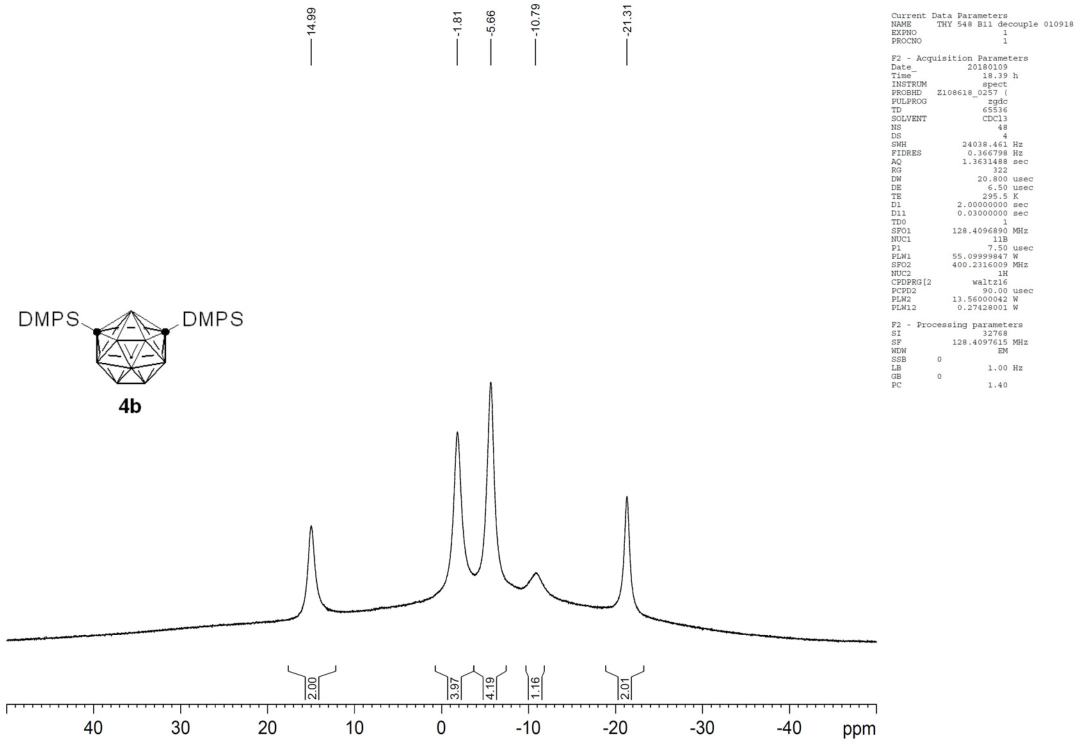

Supplementary Figure 25. $^{11}B\{^{1}H\}$ NMR spectrum of **4b** in $CDCl_3$.

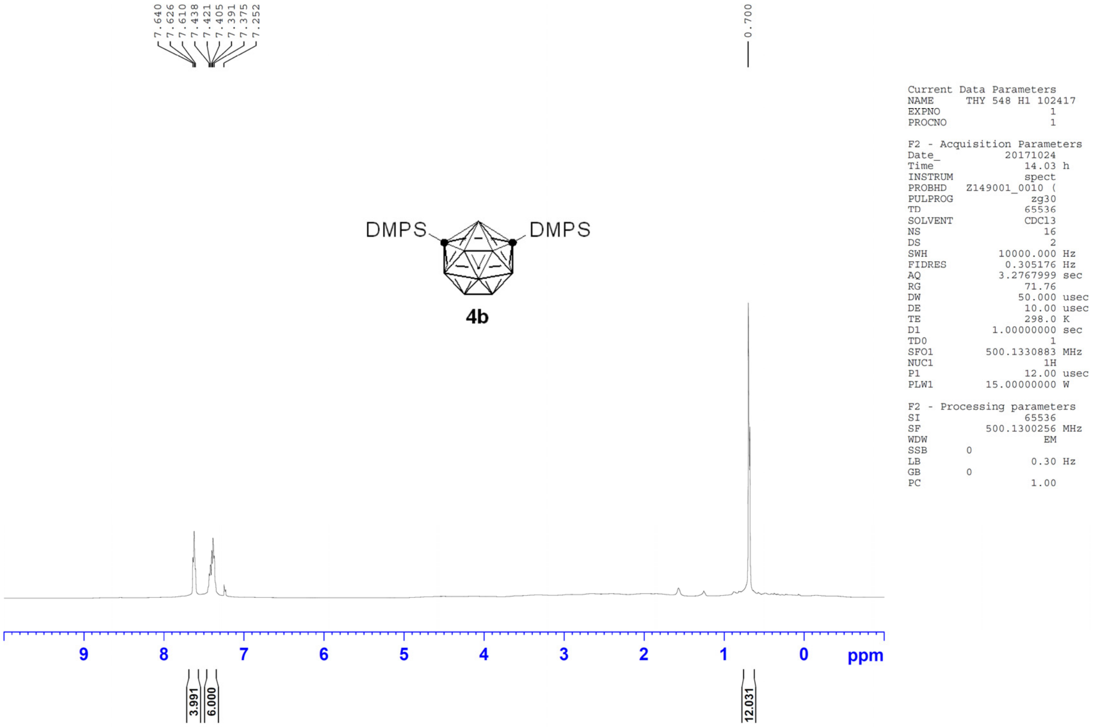

Supplementary Figure 26. ^{1}H NMR spectrum of **4b** in $CDCl_3$.

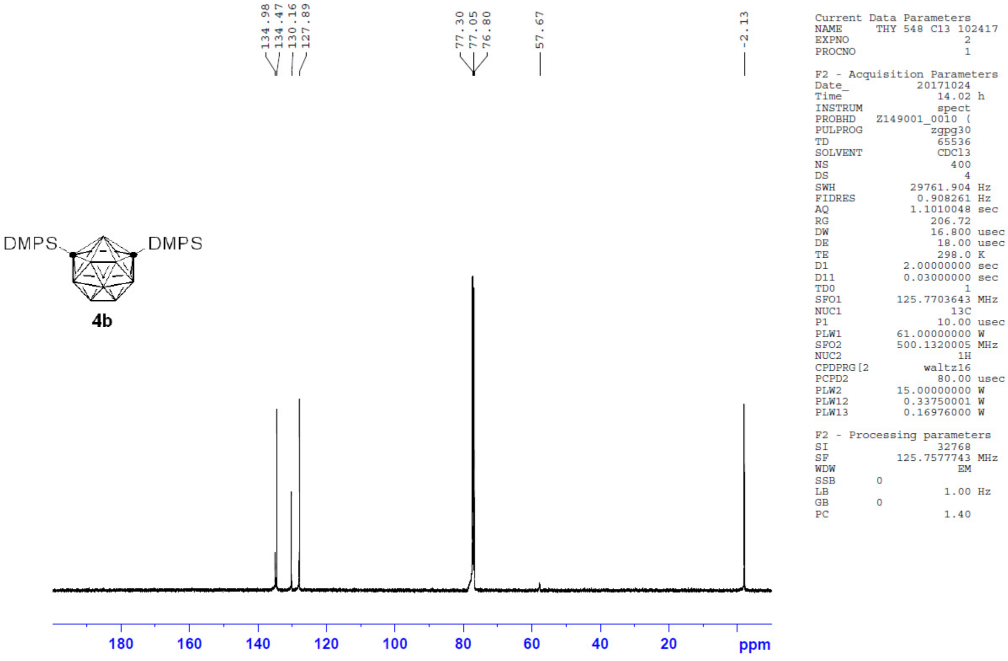

Supplementary Figure 27. ^{13}C NMR spectrum of **4b** in $CDCl_3$.

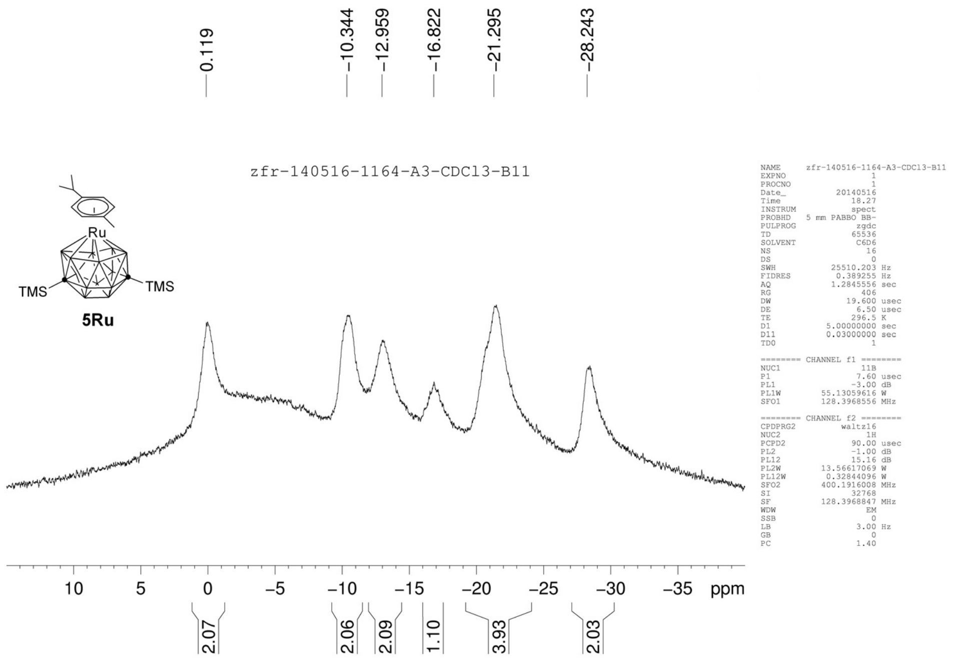

Supplementary Figure 28. $^{11}B\{^{1}H\}$ NMR spectrum of **5Ru** in $CDCl_3$.

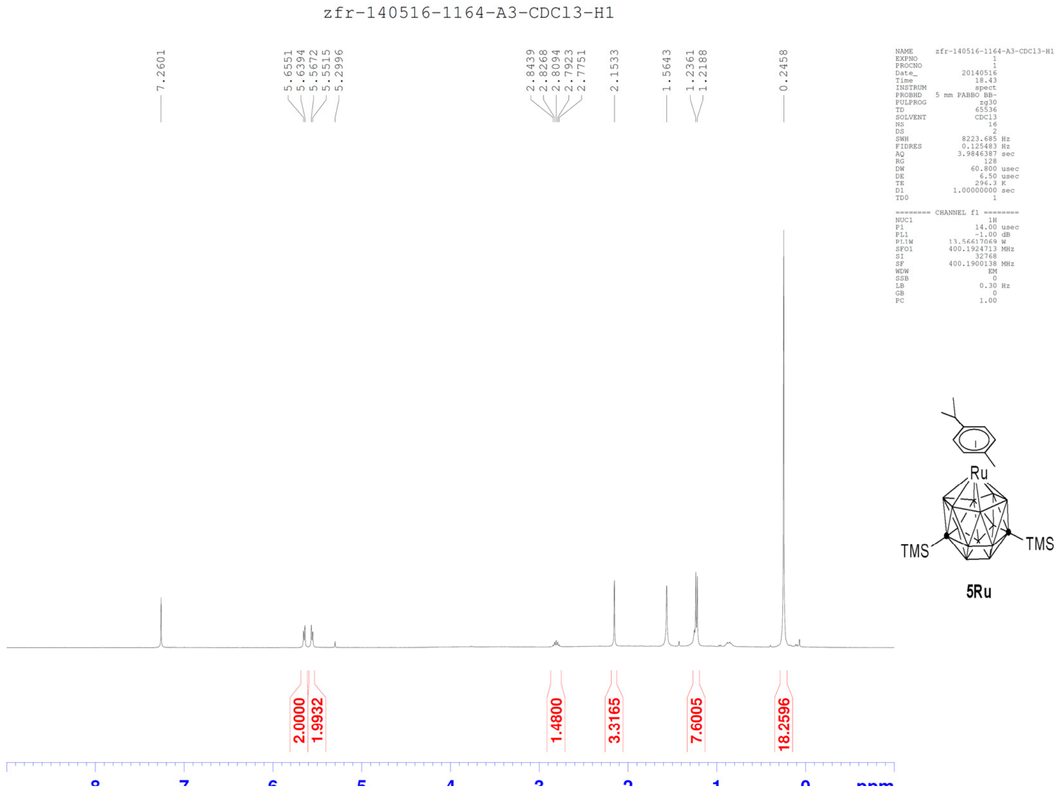

Supplementary Figure 29. ^{1}H NMR spectrum of **5Ru** in $CDCl_3$.

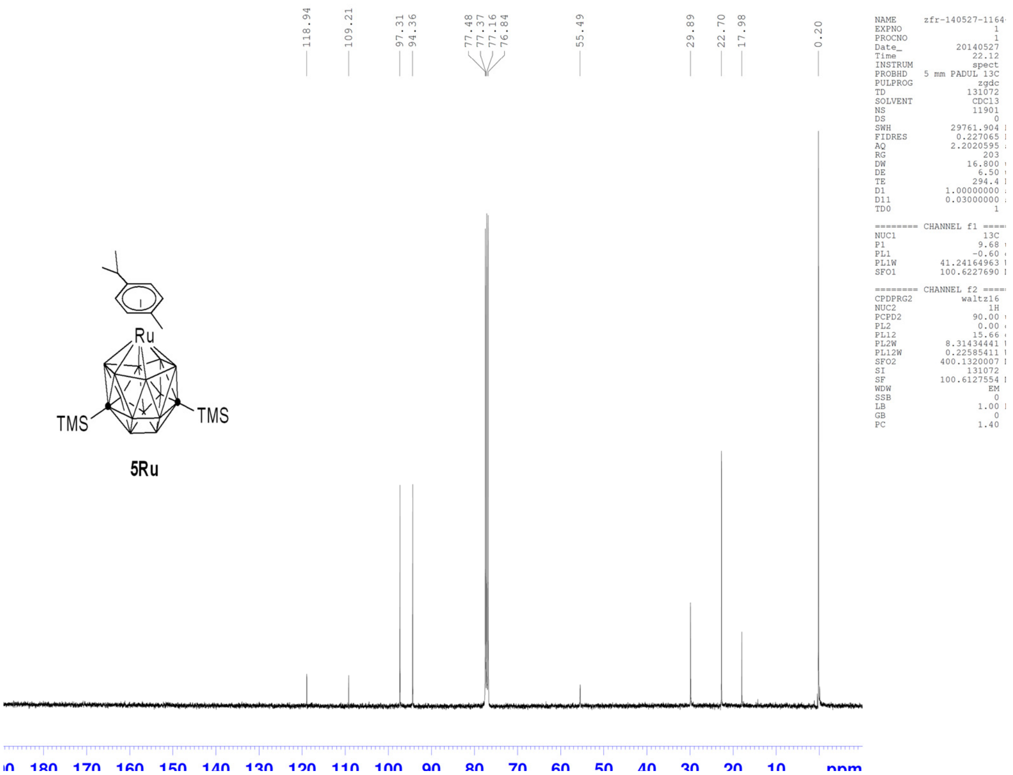

Supplementary Figure 30. ^{13}C NMR spectrum of **5Ru** in $CDCl_3$.

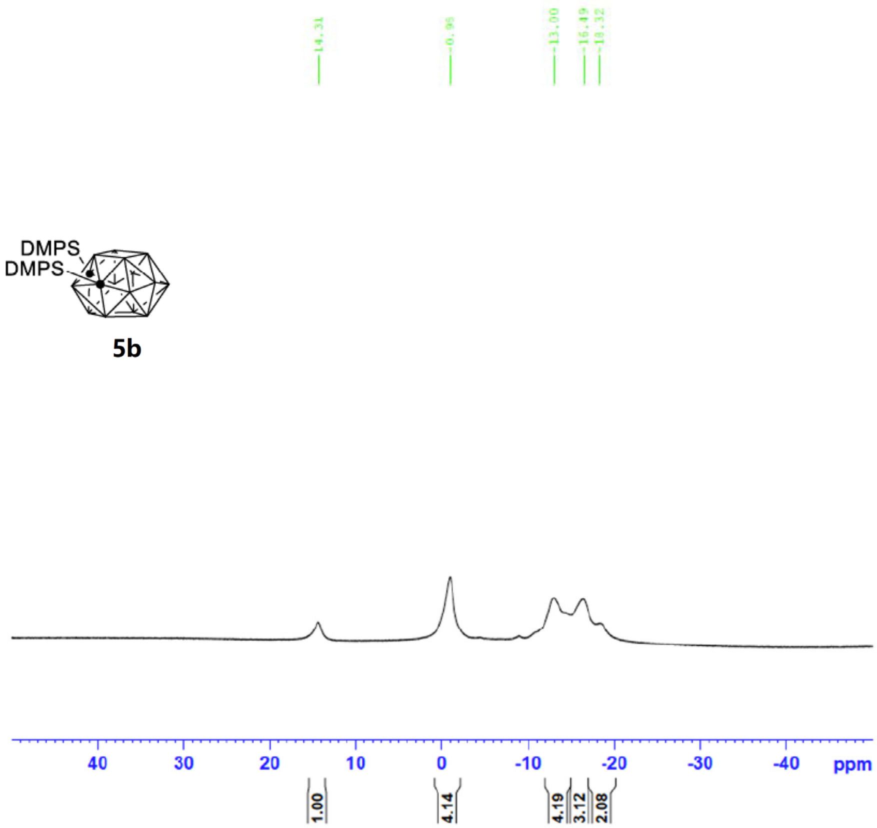

Supplementary Figure 31. $^{11}B\{^1H\}$ NMR spectrum of **5b** in $CDCl_3$.

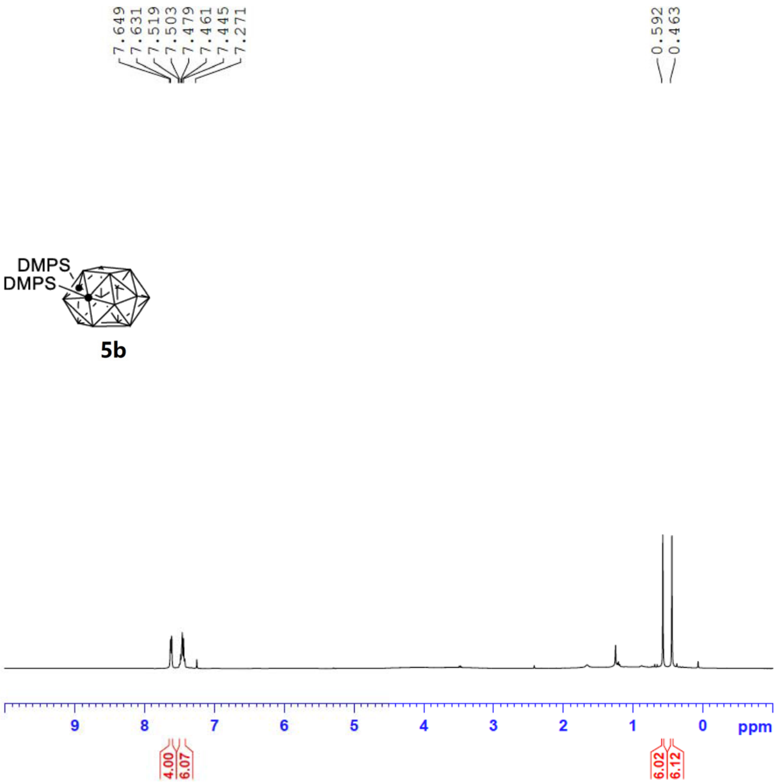

Supplementary Figure 32. ^{1}H NMR spectrum of **5b** in $CDCl_3$.

Parameter	Value
Title	THY 585 C13 020518
Spectrometer	spect
Solvent	CDCl3
Temperature	298.0
Pulse Sequence	zgpg30
Experiment	1D
Number of Scans	384
Receiver Gain	207
Relaxation Delay	2.0000
Pulse Width	10.0000
Acquisition Time	1.1010
Spectrometer Frequency	125.76
Spectral Width	29761.9
Lowest Frequency	-2294.6
Nucleus	13C
Acquired Size	32768
Spectral Size	65536

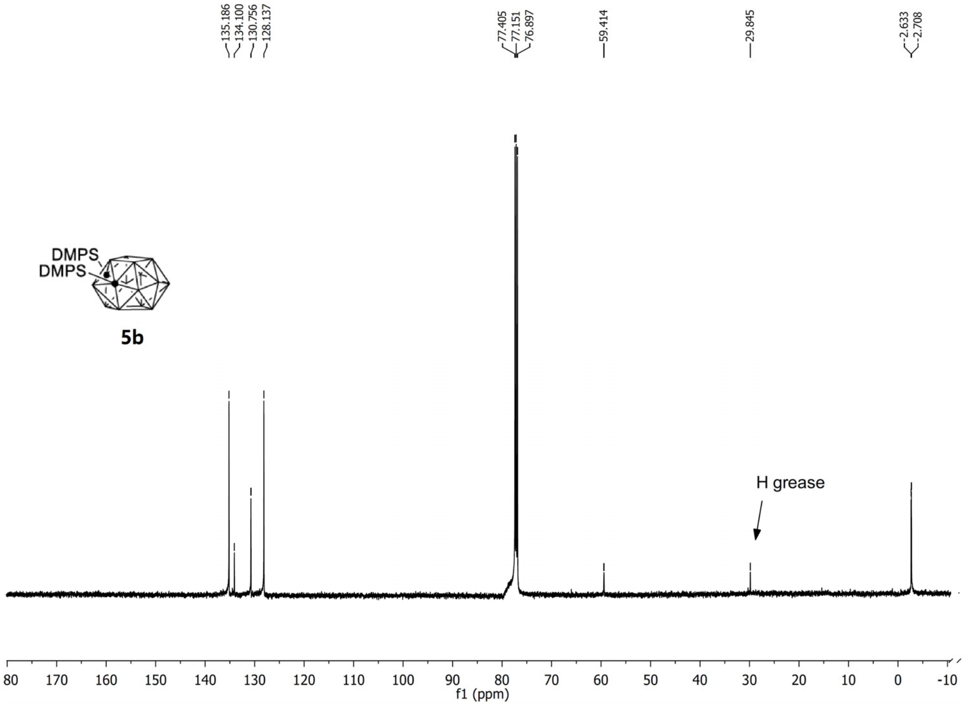

Supplementary Figure 33. ^{13}C NMR spectrum of **5b** in $CDCl_3$.

Supplementary Reference

1. Beard, C. D. & Moffitt, R. B. III. Cleavage of silylated carboranes to prepare *m*-carboranes. *U.S. Patent* 4,111,999 (1978).
2. Sheldrick, G. M. *SADABS: Program for Empirical Absorption Correction of Area Detector Data*. University of Göttingen, Germany, 1996.
3. Sheldrick, G. M. *SHELXTL 5.10 for Windows NT: Structure Determination Software Programs*. Bruker Analytical X-ray Systems, Inc., Madison, Wisconsin, USA, 1997.
4. Frisch, M. J. et al. Gaussian 09, revision D. 01; Gaussian, Inc., Pittsburgh, PA, 2009.
5. Becke, A. D. Density-functional thermochemistry. III. The role of exact exchange. *J. Chem. Phys*. **98**, 5648-5652 (1993).
6. Humphrey, W., Dalke, A. & Schulten, K. VMD: Visual molecular dynamics. *J. Mol. Graphics* **14**, 33-38 (1996)
