# Supplementary material for: Synthesis and X-ray characterization of 15- and 16-vertex closo-carboranes
Source: Nat Commun. 2020 Nov 23;11:5943. doi: 10.1038/s41467-020-19661-5 (PMC7683565; doi:10.1038/s41467-020-19661-5)
Supplement: Supplementary file 2 — Description of Additional Supplementary Files [file 41467_2020_19661_MOESM2_ESM.pdf]

Description of Additional Supplementary Files

Supplementary Data 1
Cartesian coordinates in .txt format for the optimized structure of **4b**

Supplementary Data 2
Cartesian coordinates in .txt format for the optimized structure of **5b**
